# Synthesis of Novel Derivatives of 5,6,7,8-Tetrahydroquinazolines Using α-Aminoamidines and In Silico Screening of Their Biological Activity

**DOI:** 10.3390/ijms23073781

**Published:** 2022-03-29

**Authors:** Arsenii D. Snizhko, Alexander V. Kyrychenko, Eugene S. Gladkov

**Affiliations:** 1 Institute of Chemistry and School of Chemistry, V. N. Karazin Kharkiv National University, 4 Svobody Sq., 61022 Kharkiv, Ukraine; arseniysnizko5@gmail.com (A.D.S.); a.v.kyrychenko@karazin.ua (A.V.K.); 2 State Scientific Institution “Institute for Single Crystals”, National Academy of Sciences of Ukraine, 60 Nauky Ave, 61072 Kharkiv, Ukraine

**Keywords:** α-aminoamidine, diarylidencyclohexanone, tetrahydroquinazoline, molecular docking, inhibitor, antitubercular, antidiabetic

## Abstract

α-Aminoamidines are promising reagents for the synthesis of a diverse family of pyrimidine ring derivatives. Here, we demonstrate the use of α-aminoamidines for the synthesis of a new series of 5,6,7,8-tetrahydroquinazolines by their reaction with bis-benzylidene cyclohexanones. The reaction occurs in mild conditions and is characterized by excellent yields. It has easy workup, as compared to the existing methods of tetrahydroquinazoline preparation. Newly synthesized derivatives of 5,6,7,8-tetrahydroquinazoline bear protecting groups at the C2-*tert*-butyl moiety of a quinazoline ring, which can be easily cleaved, opening up further opportunities for their functionalization. Moreover, molecular docking studies indicate that the synthesized compounds reveal high binding affinity toward some essential enzymes of *Mycobacterial tuberculosis*, such as dihydrofolate reductase (DHFR), pantothenate kinase (*Mt*PanK), and FAD-containing oxidoreductase DprE1 (*Mt*DprE1), so that they may be promising candidates for the molecular design and the development of new antitubercular agents against multidrug-resistant strains of the *Tubercle bacillus*. Finally, the high inhibition activity of the synthesized compounds was also predicted against β-glucosidase, suggesting a novel tetrahydroquinazoline scaffold for the treatment of diabetes.

## 1. Introduction

Quinazoline and tetrahydroquinazoline skeletons constitute essential structural motifs in natural products and pharmaceutically active compounds [1,2,3,4]. Recently, some 5,6,7,8-tetrahydroquinazoline derivatives have revealed antitubercular activity [5]. Therefore, developing novel routes for a synthetic design of tetrahydroquinazolines is a high-value goal, due to their promising bioactivities. Traditional approaches for preparing tetrahydroquinazoline derivatives are mainly based on the cyclocondensation of various guanidine derivatives with aldehydes and ketones, providing C2-substituted tetrahydroquinazolines [3,6,7,8,9,10,11,12]. It has been reported that various polysubstituted pyrimidines were synthesized by a base-promoted intermolecular oxidation C–N bond formation of allylic compounds with amidines, using molecular oxygen (O_2_) as the sole oxidant [13]. The suggested protocol utilized protecting-group-free nitrogen sources and good functional group tolerance. It has essential environmental advantages for green and sustainable chemistry, because molecular oxygen is known to be an ideal oxidant, due to its natural, inexpensive, and environmentally friendly characteristics [13]. 2-Methyl-tetrahydroquinazolines have been obtained with good yields (38–81%) by reacting the α,β-unsaturated ketones with acetamidine hydrochloride in acetic acid [7]. 2-Amino-tetrahydroquinazolines have been synthesized by reacting substituted diarylidencyclohexanones with guanidine hydrochloride. Unlike earlier works for the synthesis of 2-aminopyrimidines from chalcones and guanidine hydrochloride, in which an external oxidizing agent was required to convert dihydropyrimidines to pyrimidines, in the present study, the aromatization occurred by aerial oxidation [7].

Recently, this synthetic procedure was applied to a reaction of different *bis*-benzylidene cyclohexanones with guanidine hydrochloride in the presence of NaH in DMF as a solvent, resulting in the formation of 8-(arylidene)-4-(aryl)5,6,7,8-tetrahydroquinazolin-2-ylamines with a yield of 19–28% [6]. Herein, we report the use of α-aminoamidines for the synthesis of a new series of 5,6,7,8-tetrahydroquinazoline derivatives by their reaction with bis-benzylidene cyclohexanones. The method is characterized by excellent yields, mild reaction conditions, and easy workup, so that it is an advance over the existing synthetic procedures. The key advantage of new tetrahydroquinazoline derivatives is the presence of protecting groups (PG) at the C2-*tert*-butyl moiety of the quinazoline fragment. We demonstrate that these PGs can be readily cleaved, resulting in the appearance of the terminal free amino group, which opens up further opportunities for their functionalization.

Derivatives of polysubstituted pyrimidines, quinazolines, and hydroquinazolines have revealed a range of pharmacological effects, including antioxidant, antibacterial, antiviral, antifungal, antitubercular, and anti-inflammatory [12]. Therefore, the antitubercular activity of newly synthesized tetrahydroquinazoline derivatives was screened using a molecular docking approach over a series of essential enzymes of *Mycobacterial tuberculosis*, namely dihydrofolate reductase (DHFR), pantothenate kinase (*Mt* PanK), and FAD-containing oxidoreductase DprE1 (*Mt* DprE1). In addition, tetrahydroquinazolines have been reported as exhibiting inhibitory activity towards α- and β-glucosidases, which is promising for blocking specific metabolic processes involved in a variety of diseases, such as diabetes and related diseases [6]. Therefore, the in silico screening was also performed for the tetrahydroquinazoline derivatives to identify the inhibitory activities of these compounds against the *Raucaffricine* β-glucosidase. We found that some new tetrahydroquinazoline derivatives revealed high binding affinity toward all of these three key enzymes. Moreover, the molecular docking of other substances, which have recently revealed high in vitro inhibitory activity against a range of *Mycobacterium tuberculosis* strains, such as the clinically approved drugs and trial substances methotrexate, macozinone, and TBA-7371, holds promise that our ligand **3c** may reveal in vitro activity of a similar order of magnitude. Therefore, the studied ligands may also be promising candidates for the development of new antitubercular agents against multidrug-resistant strains of the *Tubercle bacillus*. Moreover, their high inhibition activity against β-glucosidase offers opportunities for the design of a novel scaffold for promising therapeutics in the treatment of diabetes.

## 2. Results and Discussion

### 2.1. Chemistry

An efficient approach for the synthesis of pyrimidine rings has been reported by using Cu-catalyzed and 4-HO-TEMPO-mediated [3 + 3] annulation of amidines with saturated ketones [14]. In this reaction, the synthesis of pyrimidines occurs by a cascade reaction of oxidative dehydrogenation/annulation/oxidative aromatization via direct β-C(sp3)−H functionalization of saturated ketones, followed by annulation with amidines [14]. In addition, an efficient method for the modular synthesis of various pyrimidine derivatives has been demonstrated by using the reactions of ketones, aldehydes, or esters with amidines catalyzed by an in situ prepared recyclable iron(II) complex [15]. It has been noticed that the reaction occurred in a regioselective fashion via a remarkable unactivated β-C−H bond functionalization [15].

**Scheme 1:**
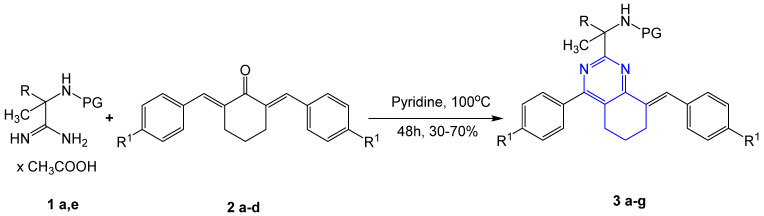
Synthesis of substituted 5,6,7,8-tetrahydroquinazolines **3a-g**.

Recently, we suggested that α-aminoamidines can be applied as substrates for subsequent transformations and synthesis of imidazole- and pyrimidine-containing building blocks yielding 21–93% [16]. Here, we further demonstrate the use of α-aminoamidines for the synthesis of novel tetrahydroquinazoline derivatives by the tuned synthetic procedure. Diarylidencyclohexanones were introduced as carbonyl derivatives instead of α,β-unsaturated ketone used in our earlier works. Using these procedures, the series of substituted 5,6,7,8-tetrahydroquinazolines **3a-g** was synthesized by the reaction of α-aminoamidines **1a,e** with diarylidencyclohexanones **2a-d** in pyridine solution by heating at 100 °C for 24 h, resulting in higher (47–80 %) yields of products **3a-g** (Scheme 1).

The mechanism of the cyclocondensation of 2-substituted pyrimidine rings in such reactions has recently been suggested as a cascade process, initiated by Michael addition of a guanidine moiety to one of the two olefinic bonds of an enone fragment of diarylidencyclohexanones [6]. A similar reaction mechanism has also been suggested by Michael addition of either acetamindine, guanidine, or phenylenediamines on one of the two olefinic bonds of the enone moiety, leading to an intermediate, followed by a reaction between the amine group and the keto group of the enone [7]. After which, upon dehydration of the dihydropyrimidine skeleton, the final cyclic products were formed [7]. These products were further aromatized by molecular oxygen converting into the target compounds [7].

The most likely outcome in our reaction conditions is that the synthesis of compounds **3a-g** would proceed through similar synthetic routes by Michael addition of α-aminoamidines **2a-d** to one of the olefinic bonds of diarylidencyclohexanones **2a-d**. All synthesized compounds are summarized in Table 1.

**Scheme 2:**
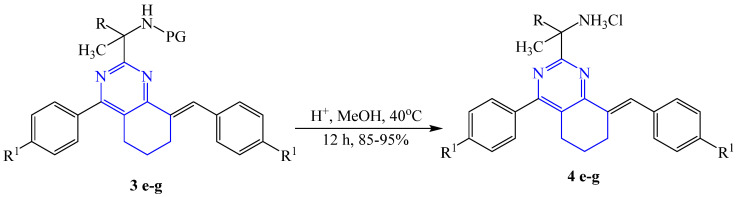
Cleavage of protecting groups in compounds **4e-g**.

Using compounds **3e-g** and the modified synthetic procedure from [10], we obtained unprotected 5,6,7,8-tetrahydroquinazolines **4e-g,** which can be used for synthetic purposes as building blocks. Cleavage of Boc-derivatives of *tert*-butyl-(2-(8-arylidene-4-aryl-5,6,7,8-tetrahydroquinazolin-2-yl)butan-2-yl)carbamates **3e-g** was realized with a good yield by stirred-in MeOH with added concentrated HCl at 40 °C for 24 h (Scheme 2).

Cleavage of the Boc-protecting group in compounds **3e-g** was proven by the absence of corresponding proton signals in the ^1^H NMR spectra, characteristic for this group, and the appearance of broadened signals of N^+^H_3_ protons (8.20–8.53 ppm) in the spectra of compounds **4e-g** (Figures S8–S10). The rest of the ^13^C NMR spectra of compounds **3a-g** and **4e-g** were assigned by signals of aromatic, aliphatic atoms and substitutes, as described in the Experimental Section and Supplementary Information (Figures S11–S20). In addition, the mass-spectra for compounds **3a-g** and **4e-g** are provided in Supplementary Information (Figures S21–S30).

### 2.2. Molecular Docking

It has recently been demonstrated that tetrahydroquinazolines are able to inhibit the activity of *Mycobacterium tuberculosis* dihydrofolate reductase (DHFR) [6]. In addition, several other enzymes have recently been identified to be involved in vital physiological functions in *Mycobacterium tuberculosis* and were suggested as novel attractive molecular targets for anti-TB drug development [17,18,19]. Among these essential mycobacterial enzymes are *M. tuberculosis* pantothenate kinase (*Mt*PanK) and *M. tuberculosis* FAD-containing oxidoreductase DprE1 (*Mt*DprE1) [20]. Therefore, the antitubercular activity of all newly synthesized compounds **3a**-**e** and **4e**-**g** were screened by molecular docking against *M. tuberculosis* DHFR (PDB code 1DF7), *Mt*PanK (PDB code 4BFT), and *Mt*DprE1 (PDB code 4FF6), respectively. The preparation of the receptors and ligands was carried out using the AutoDockTools (ADT) software, version 1.5.7 [21]. The addition of hydrogen, the calculation of the Gasteiger charges of the receptor, and ligands were also performed using the ADT software. Molecular docking calculations were performed with the AutoDock Vina 1.1.2 software [22]. During the docking, the receptor was kept rigid and the ligand molecules were conformationally flexible. In AutoDock Vina, the Lamarckian genetic algorithm was used as a research parameter. The size of the cubic box generated by ADT in the region of the DHFR receptor interaction (residues Gly15, Ile20, Gln28, Phe31, Ser49) was defined as 60 × 64 × 60 Å. The center of the grid box for the DHFR receptor was set at Cartesian coordinates x = 9309, y = 26,479, and z = 13,044. For *Mt*PanK and *Mt*DprE1 receptors, the grid box was centered at x = −18,742, y = −13.919, and z = 11,679 and x = 14,990, y=−20,507, and z = 37,226, [23] respectively. The grid-point spacing was set to 0.375 Å. The number of ligand-binding modes was set to 9 and the Vina exhaustiveness parameter to 64. For each ligand, three independent searches were performed using different random seeds. The best docking mode corresponds to the largest ligand-binding affinity.

#### 2.2.1. Molecular Docking against *Mycobacterium* *tuberculosis* Enzymes

To gain insight into the biological activity of the new tetrahydroquinazoline derivatives, their binding affinity and selectivity toward DHFR, *Mt*PanK, and *Mt*DprE1 proteins were explored and characterized using AutoDock Vina 1.1.2. We found that the compounds **3a-d** and **4e-g** had high binding affinity to DHFR, so that their Vina docking binding scores varied in the range of −9.0 ÷ −9.7 kcal/mol, as summarized in Table 2.

**Figure 1:**
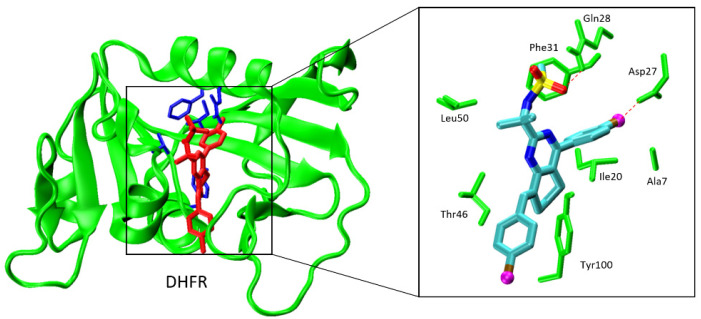
The best-scored docked pose of **3c** bound to the *Mycobacterium tuberculosis* DHFR protein (PDB code 1DF7). Ribbon representation of the structure of the DHFR-**3c** complex. The protein is shown as a ribbon model in green and **3c** is represented as a licorice model in red. The active site residues of DHFR, such as Ile5, Gln28, Phe31, Ile94, and Tyr100, are shown in blue. (Insert Panel) A scheme of protein–ligand interactions of **3c** with neighboring residues, colored green. The red dotted lines show key short-range interactions between ligand **3c** and the DHFR enzyme.

Figure 1 shows the best-scored docked pose of **3c** bound to *M. Tuberculosis* DHFR (PDB code 1DF7). The ligand occupies a long groove, largely aligned by hydrophobic residues, adjacent to the DHFR binding site, such as Phe31. Ligand **3c** was found to be well-suited within the DHFR active site, centered around residues Ile5, Gln28, Phe31, Ile94, and Tyr100, respectively [25,26,27]. In addition to several hydrophobic interactions, **3c** utilized its sulfonamide head group to interact with the protein through some hydrogen bonds by Gln28 (Figure 1*, Insert Panel*). Moreover, we identified that interactions involving active site residue Phe31 played a crucial role, driving π-π between the aromatic ring of **3c** and the neighboring Phe31 residue.

**Figure 2:**
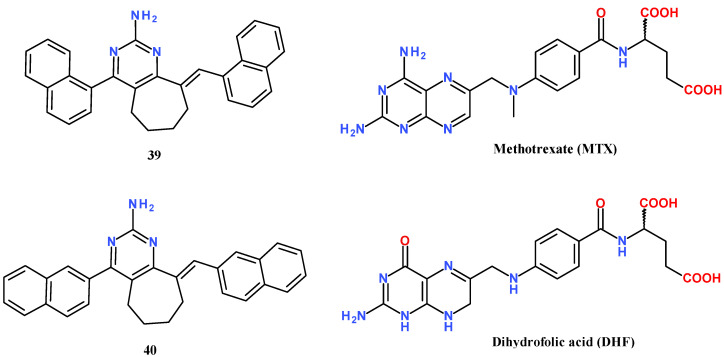

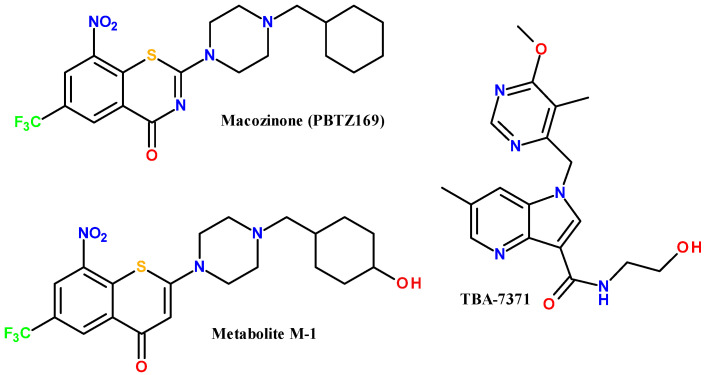
The best-docking-scored reference compounds from [6,24] and other known inhibitors for the mycobacterial DHFR, such as methotrexate (MTX), macozinone (**PBTZ169**), and **TBA-7371**. Heteroatoms and key functional groups are color-coded.

It has been reported that the DHFR groove acts as an interaction site to some structurally similar 2-aminopyrimidines, which revealed very good in vitro activity against *Mycobacterium tuberculosis* and promising docking binding scores for DHFR [6]. For these reasons, the hit molecules **39** and **40** from [6] were re-docked by AutoDock Vina to be comparable with our docking results (Figure 2 and Table 2).

It can be noticed that the most favored docking energy of −12.2 kcal/mol was found for compound **40**, possessing two naphthalene rings (Figure 2). Moreover, we also probed two other well-known inhibitors of DHFR, namely, methotrexate (MTX) and dihydrofolic acid (DHF) [28]. These inhibitors demonstrated the binding scores of −9.0 and −9.5 kcal/mol, so that we can conclude that ligands **3c** and **4g** are among the most promising antitubercular agents [29].

**Figure 3:**
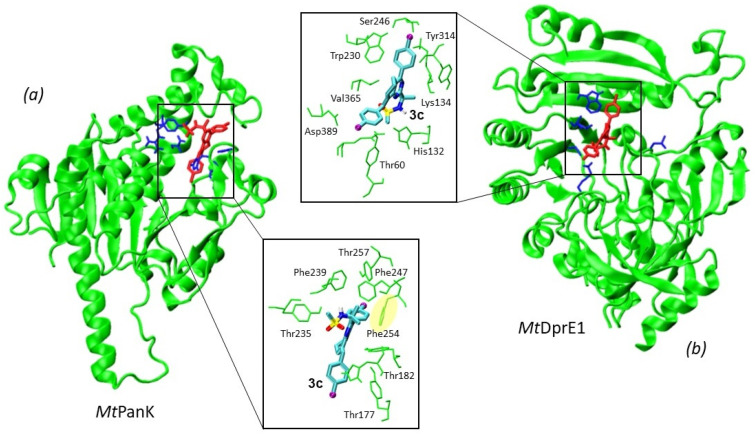
The best-scored docked pose of **3c** bound to *Mt*PanK (PDB code 4BFT) (**a**) and *Mt*DprE1 (PDB code 4FF6) (**b**), respectively. The active site residues are shown in blue as follows: *Mt*PanK —residues Tyr177, His179, Tyr282, Tyr235, Arg238, and Asn277; *Mt*DprE1—residues Ile115, Gly117, Trp230, Leu363, Val365, Cys387, and Lys418 [31], respectively. Ribbon representation of the structure of the enzyme-**3c** complex. The protein is shown as a ribbon model in green and **3c** is represented as a licorice model in red. Inserts outline the protein–ligand interactions of **3c** with neighboring enzyme residues.

Figure 3a,b shows the best-scored docked pose of **3c** bound to *Mt*PanK and *Mt*DprE1, respectively. In the case of *Mt*PanK, ligand **3c** penetrated deeply into a hydrophobic pocket of the enzyme, so that it became surrounded by aromatic side-chains of Thr177, Thr182, Phe239, Thr235, Phe247, Phe254, and Thr257, respectively (Figure 3a). The binding pose of 3c fits well within the active site of *Mt*PanK centered on Tyr177, His179, Tyr282, Tyr235, Arg238, and Asn277 [20,30], as shown in Figure 3a. The residue Phe254 plays a crucial role in the formation of the π-π stacking interaction between ligand **3c** and the *Mt*PanK protein, as seen in the insert of Figure 3a.

Recently, some novel benzothiopyranones have revealed in vitro biological activities against a range of *Mycobacterium tuberculosis* strains [24]. For these reasons, the promising metabolite M-1 from [24] and other high-potent inhibitors, such as macozinone (**PBTZ169**) and **TBA-7371** were re-docked by AutoDock Vina to be comparable with our docking results (Figure 2 and Table 2). According to the U.S. National Library of Medicine (https://ClinicalTrials.gov), **TBA-7371** (Identifier: NCT04176250) is currently at Phase 2 of the early bactericidal activity against *Pulmonary tuberculosis*. It should be noted that metabolite M-1 and the clinically trialed substance **TBA-7371** have binding scores toward *Mt*PanK in the order of −7.6–9.0 kcal/mol. Taken together, the comparison over the binding scores of the in vitro active inhibitors holds promise that our ligand **3c** may reveal the in vitro activity of a similar order of magnitude. 

Finally, inhibitory activity of the studied compounds was screened over the other important enzyme MtDprE1 of *Mycobacterium tuberculosis* strains. We found that the binding interaction of **3c** with *Mt*DprE1 was similar in many aspects to those of the *Mt*PanK enzyme (Figure 3b). Ligand **3c** binds within the active site of *Mt*DprE1, reported to be at residues Ile115, Gly117, Trp230, Leu363, Val365, Cys387, and Lys418, respectively [31]. The ligand binding is driven by hydrophobic interactions with Thr60, Trp230, and Tyr314, as shown in the insert of Figure 3b. These findings agree well with the high binding energy of **3c** equal to –10.9 kcal/mol.

#### 2.2.2. Molecular Docking against β-Glucosidase

It has been shown that tetrahydroquinazolines might inhibit glucosidases and glycogen phosphorylase enzymes [6]. Inhibition of glycoside hydrolases has essential potency in the treatment of diabetes [32,33,34]. Among the various types of glucosidase inhibitors, disaccharides, iminosugars, carbasugars, and thiosugars play an essential role [32]. However, some more effective inhibitors of glucosidases from tea polyphenol extracts, which are not based on a sugar scaffold, have recently been proposed [35,36,37,38,39]. Therefore, to gain further insight into the antidiabetic action of these compounds, molecular docking studies against *Raucaffricine* β-glucosidase (PDB code 4A3Y) were carried out and docking binding energies are also summarized in Table 2. Interestingly, the docking energy estimated through molecular docking studies was found to be in the range of −10.0 ÷ −11.1 kcal/mol. Therefore, these new compounds may represent a novel chemical scaffold for developing highly potent and specific antidiabetic drugs.

**Figure 4:**
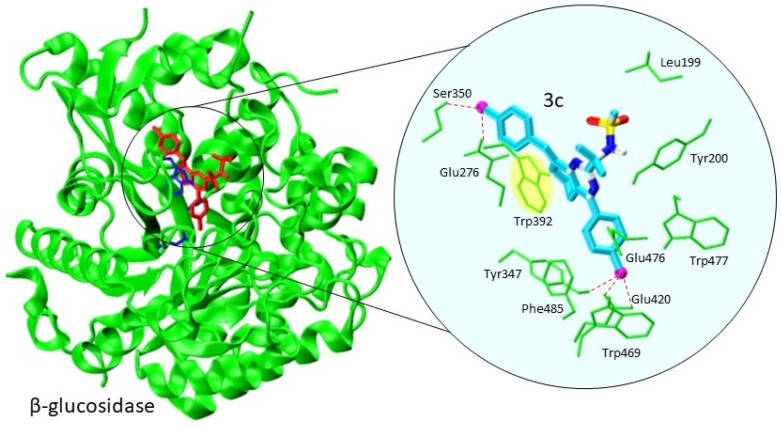
The best docking pose of **3c** at *Raucaffricine* β-glucosidase (PDB code 4A3Y). The enzyme is shown as a ribbon model in green and **3c** is represented as a licorice model in red. The active site residues Glu186, Trp392, and Glu420 of β-glucosidase are shown as blue sticks. An insert outlines protein–ligand interactions of **3c** with neighboring enzyme residues (a key Trp392 is highlighted in yellow).

Figure 4 shows the best docking pose of ligand **3c** at the β-glucosidase enzyme. It can be noted that **3c** inserted deeply into the active site cleft, so that its 4-aril moiety reached the middle of the protein fold. It has been recognized that the long active site cleft of β-glucosidase provides many water-mediated hydrogen bonds and aromatic-stacking interactions. This is why this protein pocket is believed to be a critical scaffold for efficient hydrolysis of oligosaccharides by the diverse family of β-glucosidases, so that it is commonly considered as an active target in molecular docking studies [35,36,37]. It should be pointed out that the binding of ligand **3c** was driven by hydrophobic interactions with Trp392, Trp469, Trp477, and Phe485, among which Trp392 plays a crucial role. Moreover, ligand **3c** bound well-within the active site residues Glu186, Trp392, and Glu420 of β-glucosidases, as demonstrated in Figure 4. In addition, Trp392 formed a π-π staking interaction with the quinazoline ring of **3c**, as seen in the insert of Figure 4. Finally, the peripheral polar chlorine substituents of ligand **3c** participated in H-bonding with Glu276, Ser350, Glu420, and Phe485, respectively.

**Figure 5:**
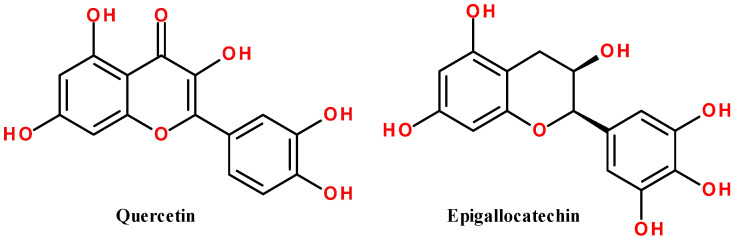
Some natural inhibitors for β-glucosidase—quercetin and epigallocatechin (**EGC**). Heteroatoms and key functional groups are color-coded.

To compare the inhibitory activity of the studied compounds, some natural inhibitors of β-glucosidase—quercetin and epigallocatechin (**EGC**)—were also screened (Table 2 and Figure 5). It should be noted that these natural inhibitors of β-glucosidase revealed binding scores of 1–2 kcal/mol smaller than those of all studied ligands (Table 2). Their docking binding scores exceeded those of some other well-known inhibitors of β-glucosidase from tea polyphenols, reported to be in the range of −7.7 ÷ −9.5 kcal/mol [35]. To sum up, it should also be noticed that the high binding affinity and regioselectivities of the studied ligands towards β-glucosidase make them promising non-sugar-based antidiabetic therapeutics.

## 3. Materials and Methods

^1^H and ^13^C NMR spectra (400 and 100 MHz, respectively) were recorded on Bruker Avance 400 and Varian MR-400 spectrometers in DMSO-*d*_6_. ^1^H and ^13^C chemical shifts were reported relative to residual protons and carbon atoms of the solvent (2.49 and 39.5 ppm, respectively) as the internal standard. The LCMS spectra were recorded using a chromatography/mass spectrometric system that consists of a high-performance liquid chromatography Agilent 1100 Series equipped with a diode matrix and a mass selective detector Agilent LC/MSD SL, column SUPELCO Ascentis Express C18 2.7 μm 4.6 mm × 15 cm. Elemental analysis was realized on a EuroVector EA-3000 instrument. TLC was performed using Polychrom SI F254 plates. Melting points of all synthesized compounds were determined with a Gallenkamp melting point apparatus in open capillary tubes.

All the starting materials were provided by Enamine Ltd. (Ukraine). According to the HPLC MS data, all synthesized compounds are >95% pure. Preliminary spectral analysis was also provided by Enamine Ltd. (Ukraine). All solvents and reagents were commercial grade and, if required, purified in accordance with the standard procedures. Precursor α-aminoamidines **1a,e** were synthesized as described elsewhere [16]. Diarylidenecyclohexanones **2a**-**d** were prepared as described elsewhere [40].

### 3.1. General Procedure for the Preparation of Protected 2-(5,6,7,8-Tetrahydroquinazolin-2-yl)propan-2-amines ***3a-g***

Corresponding protected α-aminoamidine acetates **1a,e** (1 mmol) and diarylidencyclohexanone (1 mmol) were dissolved in pyridine (15 mL). The mixture was heated at 100 °C for 24 h. After completion of the reaction (TLC control), the solvent was removed under vacuum, methanol (20 mL) was added, and the mixture was cooled to 0 °C. The crude residue of **3a-g** was filtered and washed with methanol (20 mL).

#### 3.1.1. *N*-(2-(8-Benzylidene-4-phenyl-5,6,7,8-tetrahydroquinazolin-2-yl)propan-2-yl)methanesulfonamide (**3a**)

Yield 0.30 g (70%), yellow solid, mp 142–143 °C. ^1^H NMR spectrum, δ, ppm: 8.29 (s, 1H, NH), 7.65 (d, *J* = 6.5 Hz, 2H, Ar), 7.48 (d, *J* = 6.3 Hz, 5H, Ar), 7.45–7.38 (m, 3H, Ar), 7.31 (t, *J* = 7.4 Hz, 1H, CH), 2.86 (t, *J* = 5.7 Hz, 2H, CH_2_), 2.81 (br. s, 5H, SO_2_CH_3_, and CH_2_), 1.69 (m, 7H, 2CH_3_, and CH_2_). ^13^C NMR spectrum, δ, ppm: 169.0, 165.3, 159.7, 138.4, 134.5, 130.6, 130.2, 129.7, 129.6, 128.9, 128.7, 128.6, 128.2, 124.9, 60.7, 44.3, 29.1, 27.6, 27.0, 22.5. Mass spectrum, *m*/*z* (*I*_rel_, %): 434.2 [M+H]^+^(100). Found, %: C 69.30; H 6.22; N 9.70. C_25_H_27_N_3_O_2_S. Calculated, %: C 69.26; H 6.28; N 9.69.

#### 3.1.2. *N*-(2-(8-(4-Methoxybenzylidene)-4-(4-methoxyphenyl)-5,6,7,8-tetrahydroquinazolin-2-yl)propan-2-yl)methanesulfonamide (**3b**)

Yield 0.32 g (65%), yellow solid, mp 143–144 °C. ^1^H NMR spectrum, δ, ppm: 8.25 (s, 1H, NH), 7.68 (d, *J* = 8.3 Hz, 2H, Ar), 7.48 (d, *J* = 8.5 Hz, 2H, Ar), 7.42 (s, 1H, CH), 7.06 (d, *J* = 8.5 Hz, 2H, Ar), 7.01 (d, *J* = 8.1 Hz, 2H, Ar), 3.83 (s, 3H, OCH_3_), 3.80 (s, 3H, OCH_3_), 2.86 (m, 4H, 2CH_2_), 2.82 (s, 3H, SO_2_CH_3_), 1.70 (br. s, 8H, 2CH_3_, and CH_2_). ^13^C NMR spectrum, δ, ppm: 168.7, 164.5, 160.6, 159.3, 132.7, 131.9, 131.3, 130.6, 130.2, 129.5, 124.3, 114.6, 114.4, 114.0, 60.6, 55.7, 44.2, 29.1, 27.7, 27.3, 22.6. Mass spectrum, *m*/*z* (*I*_rel_, %): 494.2 [M+H]^+^(100). Found, %: C 65.65; H 6.30; N 8.43. C_27_H_31_N_3_O_4_S. Calculated, %: C 65.70; H 6.33; N 8.51.

#### 3.1.3. *N*-(2-(8-(4-Chlorobenzylidene)-4-(4-chloromethoxyphenyl)-5,6,7,8-tetrahydroquinazolin-2-yl)propan-2-yl)methanesulfonamide (**3c**)

Yield 0.25 g (50%), yellow solid, mp 201–202 °C. ^1^H NMR spectrum, δ, ppm: 8.26 (s, 1H, NH), 7.71 (d, *J* = 8.3 Hz, 2H, Ar), 7.56 (d, *J* = 8.4 Hz, 2H, Ar), 7.49 (m, 4H, Ar), 7.43 (s, 1H, CH), 2.83 (m, 4H, 2CH_2_), 2.80 (s, 3H, SO_2_CH_3_), 1.68 (br. s, 8H, 2 CH_3_, and CH_2_). ^13^C NMR spectrum, δ, ppm: 168.8, 163.6, 159.2, 136.6, 135.3, 134.7, 134.2, 132.3, 131.5, 131.1, 128.9, 128.5, 128.2, 124.6, 60.2, 43.8, 28.5, 27.0, 26.4, 21.9. Mass spectrum, *m*/*z* (*I*_rel_, %): 502.0 [M+H]^+^(100). Found, %: C 59.81; H 5.05; N 8.31. C_25_H_25_Cl_2_N_3_O_2_S. Calculated, %: C 59.76; H 5.02; N 8.36.

#### 3.1.4. *N*-(2-(8-(4-Nitrobenzylidene)-4-(4-nitrophenyl)-5,6,7,8-tetrahydroquinazolin-2-yl)propan-2-yl)methanesulfonamide (**3d**)

Yield 0.42 g (80%), yellow solid, mp 162–163 °C. ^1^H NMR spectrum, δ, ppm: 8.40 (s, 1H, NH), 8.34 (d, *J* = 8.3 Hz, 2H, Ar), 8.27 (d, *J* = 8.4 Hz, 2H, Ar), 7.95 (d, *J* = 8.4 Hz, 2H, Ar), 7.76 (d, *J* = 8.4 Hz, 2H, Ar), 7.52 (s, 1H, CH), 2.96–2.75 (m, 7H, 2 CH_2_, and SO_2_CH_3_), 1.71 (br. s, 8H, 2 CH_3_, and CH_2_). ^13^C NMR spectrum, δ, ppm: 169.6, 163.7, 159.4, 148.3, 146.6, 144.4, 143.7, 137.9, 131.3, 131.1, 128.7, 125.9, 124.0, 123.8, 60.7, 44.4, 28.9, 27.5, 26.6, 22.2. Mass spectrum, *m*/*z* (*I*_rel_, %): 524.0 [M+H]^+^(100). Found, %: C 57.30; H 4.75; N 13.38. C_25_H_25_N_5_O_6_S. Calculated, %: C 57.35; H 4.81; N 13.38.

#### 3.1.5. *tert*-Butyl-(2-(8-benzylidene-4-phenyl-5,6,7,8-tetrahydroquinazolin-2-yl)butan2-yl)carbamate (**3e**)

Yield 0.27 g (58%), yellow solid, mp 137–139 °C. ^1^H NMR spectrum, δ, ppm: 8.17 (s, 1H, CH), 7.60 (m, 2H, Ar), 7.53–7.38 (m, 7H, Ar), 7.31 (t, 1H, Ar), 6.91 (br. s, 1H, NH), 2.85 (t, 2H, CH_2_), 2.78 (t, 2H, CH_2_), 2.05 (m, 2H, CH_2_), 1.65 (m, 2H, CH_2_), 1.59 (s, 3H, CH_3_), 1.32 (br. s, 9H, OC(CH_3_)_3_), 0.68 (t, *J* = 7.3 Hz, 3H, CH_3_). ^13^C NMR spectrum, δ, ppm: 165.2, 163.7, 159.5, 138.5, 136.9, 134.8, 130.7, 130.1, 130.0, 129.6, 129.4, 128.9, 128.6, 128.3, 128.1, 77.8, 60.4, 28.7, 27.6, 26.9, 25.5, 8.8. Mass spectrum, *m*/*z* (*I*_rel_, %): 470.4 [M+H]^+^(100). Found, %: C 76.70; H 7.50; N 8.98. C_30_H_35_N_3_O_2_. Calculated, %: C 76.73; H 7.51; N 8.95.

#### 3.1.6. *tert*-Butyl-(2-(8-(4-methoxybenzylidene)-4-(4-methoxyphenyl)-5,6,7,8tetrahydroquinazolin-2-yl)butan-2-yl)carbamate (**3f**)

Yield 0.25 g (47%), yellow solid, mp 141–142 °C. ^1^H NMR spectrum, δ, ppm: 8.10 (s, 1H, CH), 7.59 (d, *J* = 8.4 Hz, 2H, Ar), 7.42 (d, *J* = 8.4 Hz, 2H, Ar), 7.03 (d, *J* = 8.5 Hz, 2H, Ar), 6.98 (d, *J* = 8.5 Hz, 2H, Ar), 6.91 (br. s, 1H, NH), 3.80 (s, 3H, OCH_3_), 3.77 (s, 3H, OCH_3_), 2.82 (m, 4H, 2 CH_2_), 2.19–1.92 (m, 2H, CH_2_), 1.65 (m, 2H, CH_2_), 1.59 (s, 3H, CH_3_), 1.33 (br. s, 9H, OC(CH_3_)_3_), 0.65 (t, *J* = 7.1 Hz, 3H, CH_3_). ^13^C NMR spectrum, δ, ppm: 165.5, 163.5, 159.3, 136.3, 132.8, 131.8, 131.2, 129.7, 129.4, 121.6, 120.0, 115.3, 114.5, 114.0, 109.4, 102.6, 77.6, 55.7, 28.7, 27.7, 27.1, 25.6, 8.8. Mass spectrum, *m*/*z* (*I*_rel_, %): 530.2 [M+H]^+^(100). Found, %: C 72.54; H 7.47; N 7.90. C_32_H_39_N_3_O_4_. Calculated, %: C 72.56; H 7.42; N 7.93.

#### 3.1.7. *tert*-Butyl-(2-(8-(4-chlorobenzylidene)-4-(4-chlorophenyl)-5,6,7,8tetrahydroquinazolin-2-yl)butan-2-yl)carbamate (**3g**)

Yield 0.29 g (54%), yellow solid, mp 184–185 °C. ^1^H NMR spectrum, δ, ppm: 8.12 (s, 1H, CH), 7.64 (d, *J* = 8.3 Hz, 2H, Ar), 7.55 (d, *J* = 8.3 Hz, 2H, Ar), 7.47 (s, 4H, Ar), 6.90 (br. s, 1H, NH), 2.80 (m, 4H, 2 CH_2_), 2.05 (m, 2H, CH_2_), 1.66 (m, 2H, CH_2_), 1.58 (s, 3H, CH_3_), 1.30 (br. s, 9H, OC(CH_3_)_3_), 0.68 (t, *J* = 7.4 Hz, 3H, CH_3_). ^13^C NMR spectrum, δ, ppm: 164.0, 163.2, 159.1, 137.2, 135.5, 134.6, 132.7, 131.8, 131.4, 129.0, 128.7, 127.7, 124.6, 77.8, 60.4, 28.7, 27.5, 26.8, 25.4, 8.8. Mass spectrum, *m*/*z* (*I*_rel_, %): 538.2 [M+H]^+^(100). Found, %: C 66.90; H 6.13; N 7.82. C_30_H_33_N_3_O_2_Cl_2_. Calculated, %: C 66.91; H 6.18; N 7.80.

### 3.2. General Procedure for the Preparation of 2-(8-Aryliden-4-aryl-5,6,7,8-tetrahydroquinazolin-2-yl)butan-2-amine hydrochloride ***4e-g***

*tert*-Butyl-(2-(8-arylidene-4-aryl-5,6,7,8-tetrahydroquinazolin-2-yl)butan-2-yl)carbamate **3e-g** (100 mg) was mixed with MeOH (20 mL), and concentrated HCl (2 mL) was added to the solution. The reaction mixture was stirred at 40 °C for 24 h. After that, solvent was removed under reduced pressure. The solid was rinsed with dry MeCN and then dried under vacuum.

#### 3.2.1. 2-(8-(4-Benzylidene)-4-(4-phenyl)-5,6,7,8-tetrahydroquinazolin-2-yl)butan-2-amine hydrochloride (**4e**)

Yield 0.075 g (88%), white solid, mp 159–160 °C. 1H NMR spectrum, δ, ppm: 8.50 (br. s, 3H, N^+^H_3_), 8.37 (s, 1H, Ar), 7.69 (m, 2H, Ar), 7.52 (m, 5H, Ar), 7.44 (t, *J* = 7.5 Hz, 2H, Ar), 7.33 (t, *J* = 7.2 Hz, 1H, Ar), 2.85 (m, 4H, 2 CH_2_), 2.14–1.90 (m, 2H, CH_2_), 1.66 (m, 5H, CH_2_, and CH_3_), 0.80 (t, *J* = 7.4 Hz, 3H, CH_3_). 13C NMR spectrum, δ, ppm: 166.7, 164.8, 160.3, 137.9, 137.0, 133.7, 130.4, 130.0, 129.8, 128.8, 128.7, 128.3, 125.8, 61.2, 32.7, 27.5, 27.1, 24.3, 22.4, 8.5. Mass spectrum, *m*/*z* (*I*rel, %): 370.4 [M+H]^+^(100). Found, %: C 73.99; H 6.93; N 10.40. C_25_H_27_N_3_·HCl. Calculated, %: C 73.96; H 6.95; N 10.35.

#### 3.2.2. 2-(8-(4-Methoxybenzylidene)-4-(4-methoxyphenyl)-5,6,7,8-tetrahydroquinazolin-2-yl)butan-2-amine hydrochloride (**4f**)

Yield 0.08 g (92%), white solid, mp 169–170 °C. 1H NMR spectrum, δ, ppm: 8.32 (s, 1H, CH), 8.20 (br. s, 3H, N^+^H_3_), 7.70 (d, 2H, Ar), 7.50 (d, 2H, Ar), 7.05 (d, 2H, Ar), 7.00 (d, 2H, Ar), 3.80 (s, 3H, OCH_3_), 3.77 (s, 3H, OCH_3_), 2.86 (m, 4H, 2CH_2_), 2.12–1.93 (m, 2H, CH_2_), 1.65 (m, 5H, CH_2_, and CH_3_), 0.79 (t, *J* = 7.4 Hz, 3H, CH_3_). 13C NMR spectrum, δ, ppm: 164.6, 160.8, 159.4, 132.1, 131.6, 130.2, 129.5, 125.1, 114.4, 114.0, 61.1, 55.7, 32.7, 27.7, 24.3, 22.5, 8.5. Mass spectrum, *m*/*z* (*I*rel, %): 430.2 [M+H]^+^(100). Found, %: C 69.62; H 6.92; N 9.05. C_27_H_31_N_3_O_2_·HCl. Calculated, %: C 69.59; H 6.92; N 9.02.

#### 3.2.3. 2-(8-(4-Chlorobenzylidene)-4-(4-chlorophenyl)-5,6,7,8-tetrahydroquinazolin-2-yl)butan-2-amine hydrochloride (**4g**)

Yield 0.08 g (95%), white solid, mp 276–277 °C. 1H NMR spectrum, δ, ppm: 8.50 (br. s, 3H, N^+^H_3_), 8.35 (s, 1H, CH), 7.74 (d, *J* = 8.3 Hz, 2H, Ar), 7.59 (d, *J* = 8.3 Hz, 2H, Ar), 7.55 (d, *J* = 8.5 Hz, 2H, Ar), 7.50 (d, *J* = 8.4 Hz, 2H, Ar), 2.85 (m, 4H, 2 CH_2_), 2.13–1.94 (m, 2H, CH_2_), 1.67 (m, 5H, CH_2_, and CH_3_), 0.79 (t, *J* = 7.5 Hz, 3H, CH_3_). 13C NMR spectrum, δ, ppm: 164.8, 158.2, 136.6, 135.7, 135.1, 133.0, 132.1, 131.7, 130.7, 128.9, 128.8, 126.1, 61.2, 32.7, 27.4, 26.9, 24.3, 22.3, 8.5. Mass spectrum, *m*/*z* (*I*rel, %): 438.2 [M+H]^+^(100). Found, %: C 63.20; H 5.49; N 8.86. C_25_H_25_N_3_Cl_2_·HCl. Calculated, %: C 63.24; H 5.52; N 8.85.

## 4. Conclusions

Tetrahydroquinazoline scaffolds have attracted considerable interest as valuable building blocks in organic synthesis and because they are widely found in natural products that exhibit a wide range of multifunctional biological activities [41,42]. By using *bis*-benzylidene cyclohexanones and α-aminoamidines as nitrogen sources, a versatile and practical protocol for the synthesis of diversely substituted 5,6,7,8-tetrahydroquinazolines is provided. One of the advantages of newly synthesized derivatives **3a**-**g** is that they bear protecting groups at the C2-*tert*-butyl moiety of a quinazoline ring. The latter can be easily cleaved, opening up further opportunities for the functionalization of a tetrahydroquinazoline scaffold. To probe the biological activities of the new tetrahydroquinazoline derivatives, we carried out molecular docking against some essential enzymes of *Mycobacterial tuberculosis*, such as DHFR, *Mt*PanK, and *Mt*DprE1, respectively. Analysis of the binding energy scores and protein–ligand interaction maps indicated that some derivatives, such as **3c** and **3d**, revealed promising characteristics of inhibitor–enzyme docking, so that they may be attractive candidates for the molecular design of new antitubercular drugs against multidrug-resistant strains of the *Tubercle bacillus*. We also believe that these high docking scores evaluated using identical docking parameters hold a promise that our ligand **3c** may reveal the in vitro activity of a similar order of magnitude as the existing well-known inhibitors. In addition, the literature analysis suggests that the synthesized tetrahydroquinazoline derivatives may exhibit multifunctional inhibitory activity against various receptors. Therefore, we plan to address this matter in detail once all in silico screenings have been performed.

Moreover, molecular docking studies of compounds **3a-g** and **4e-g** against *Raucaffricine* β-glucosidase revealed high inhibition potency, with the binding energy score being in the range of −10.0 ÷ −11.1 kcal/mol, making them a promising chemical scaffold for developing antidiabetic drugs.

## Figures and Tables

**Table 1:** Reagents **1a,e**, **2a-d,** and synthesized products **3a-g** and **4e-g**.

Compounds	Protecting Group (PG)	R	R^1^	Yields, %
**1a**	Ms	Me	-	-
**1e**	Boc	Et	-	-
**2a**	-	-	H	-
**2b**	-	-	OCH_3_	-
**2c**	-	-	Cl	-
**2d**	-	-	NO_2_	-
**3a**	Ms	Me	H	70
**3b**	Ms	Me	OCH_3_	65
**3c**	Ms	Me	Cl	50
**3d**	Ms	Me	NO_2_	80
**3e**	Boc	Et	H	57
**3f**	Boc	Et	OCH_3_	47
**3g**	Boc	Et	Cl	54
**4e**	H	Et	H	88
**4f**	H	Et	OCH_3_	92
**4g**	H	Et	Cl	95

^1^H NMR spectra of compounds **3a-g** are characterized by signals of NH protons at 7.43–7.48 ppm (for Ms-protected derivatives **3a-d** (Figures S1–S4)) and 6.90–6.97 ppm (for Boc-protected derivatives **3e-g** (Figures S5–S7)), aromatic protons of two aryl cycles, singlets of CH=proton at 8.17–8.50 ppm, and aliphatic protons at around 2.90 ppm (4H) and 1.70 ppm (2H); and proton protecting group and protons of substitutes (Figures S1–S7).

**Table 2:** Molecular docking binding score for **3a-d**, **4e-g,** and some existing inhibitors toward the key enzymes of *Mycobacterium tuberculosis* and β-glucosidase.

Compounds	M_w_ (g/mol) ^1^	Docking Binding Energy (kcal/mol)				logP ^2^
		*Mycobacterium Tuberculosis* DHFR (PDB 1DF7)	Pantothenate Kinase (*M*tPanK) (PDB 4BFT)	FAD-Containing Oxidoreductase (*Mt*DprE1) (PDB 4FF6)	β-Glucosidase (PDB 4A3Y)	
**studied ligands**						
**3a**	433.6	−9.3	−9.7	−10.6	−10.3	4.61
**3b**	493.6	−9.0	−9.4	−10.9	−10.7	4.47
**3c**	523.6	−9.6	−9.6	−10.9	−10.9	5.82
**3d**	502.5	−9.4	−9.4	−10.4	−11.1	2.77
**4e**	369.5	−9.1	−9.1	−9.1	−10.6	4.78
**4f**	429.6	−9.3	−8.8	−9.1	−10.0	4.64
**4g**	438.4	−9.7	−9.6	−8.5	−10.3	5.99
**reference inhibitors**						
**39** from [6]	427.6	−11.2	−10.7	-^3^	−12.5	6.98
**40** from [6]	427.6	−12.2	−11.0	−11.1	−12.5	6.98
methotrexate (MTX)	454.5	−9.0	−8.5	−9.4	−8.7	−1.23
dihydrofolic acid (DHF)	443.4	−9.5	−9.2	−9.7	−9.1	−2.16
macozinone (**PBTZ169**)	456.5	−10.3	−8.8	−9.8	−9.2	4.42
**M-1** from [24]	471.5	−10.4	−9.0	−9.8	−9.5	3.56
**TBA-7371**	355.4	−8.3	−7.6	−8.4	−8.4	1.31
quercetin	302.2	−8.4	−8.3	−9.1	−9.2	0.35
epigallocatechin (**EGC**)	306.3	−8.1	−7.8	−8.1	−8.8	1.11

^1^ For 4**e**-**f,** M_w_ is given for free base. ^2^ LogP was calculated by OSIRIS Property Explorer, available at http://www.organic-chemistry.org/prog/peo/. ^3^ The best docking binding of compound **39** to *Mt*DprE1 occurred outside of the active site of the enzyme.

## Supplementary Materials

The following supporting information can be downloaded at: https://www.mdpi.com/article/10.3390/ijms23073781/s1.

## References

[1] Foster B.A., Coffey H.A., Morin M.J., Rastinejad F. (1999). Pharmacological rescue of mutant p53 conformation and function. Science.

[2] Henderson E.A., Bavetsias V., Theti D.S., Wilson S.C., Clauss R., Jackman A.L. (2006). Targeting the α-folate receptor with cyclopenta[g]quinazoline-based inhibitors of thymidylate synthase. Bioorg. Med. Chem..

[3] Kumar A., Pasam V.R., Thakur R.K., Singh M., Singh K., Shukla M., Yadav A., Dogra S., Sona C., Umrao D. (2019). Novel tetrahydroquinazolinamines as selective histamine 3 receptor antagonists for the treatment of obesity. J. Med. Chem..

[4] Ortega J.A., Arencibia J.M., Minniti E., Byl J.A.W., Franco-Ulloa S., Borgogno M., Genna V., Summa M., Bertozzi S.M., Bertorelli R. (2020). Novel, potent, and druglike tetrahydroquinazoline inhibitor that is highly selective for human topoisomerase ii α over β. J. Med. Chem..

[5] Yan M., Ma S. (2012). Recent advances in the research of heterocyclic compounds as antitubercular agents. ChemMedChem.

[6] Singh N., Pandey S.K., Anand N., Dwivedi R., Singh S., Sinha S.K., Chaturvedi V., Jaiswal N., Srivastava A.K., Shah P. (2011). Synthesis, molecular modeling and bio-evaluation of cycloalkyl fused 2-aminopyrimidines as antitubercular and antidiabetic agents. Bioorg. Med. Chem. Lett..

[7] El-Subbagh H.I., Hassan G.S., El-Messery S.M., Al-Rashood S.T., Al-Omary F.A.M., Abulfadl Y.S., Shabayek M.I. (2014). Nonclassical antifolates, part 5. Benzodiazepine analogs as a new class of dhfr inhibitors: Synthesis, antitumor testing and molecular modeling study. Eur. J. Med. Chem..

[8] Rong L., Han H., Wang H., Jiang H., Tu S., Shi D. (2009). An efficient and facile synthesis of pyrimidine and quinazoline derivatives via one-pot three-component reaction under solvent-free conditions. J. Heterocycl. Chem..

[9] Zakeri M., Nasef M.M., Abouzari-Lotf E. (2014). Eco-safe and expeditious approaches for synthesis of quinazoline and pyrimidine-2-amine derivatives using ionic liquids aided with ultrasound or microwave irradiation. J. Mol. Liq..

[10] Al-Omary F.A.M., Hassan G.S., El-Messery S.M., El-Subbagh H.I. (2012). Substituted thiazoles v. Synthesis and antitumor activity of novel thiazolo[2,3-b]quinazoline and pyrido[4,3-d]thiazolo[3,2-a]pyrimidine analogues. Eur. J. Med. Chem..

[11] Gao Q., Wu M., Zhang K., Yang N., Liu M., Li J., Fang L., Bai S., Xu Y. (2020). I2-catalyzed aerobic α,β-dehydrogenation and deamination of tertiary alkylamines: Highly selective synthesis of polysubstituted pyrimidines via hidden acyclic enamines. Org. Lett..

[12] Rashid H.u., Martines M.A.U., Duarte A.P., Jorge J., Rasool S., Muhammad R., Ahmad N., Umar M.N. (2021). Research developments in the syntheses, anti-inflammatory activities and structure–activity relationships of pyrimidines. RSC. Adv..

[13] Guo W., Li C., Liao J., Ji F., Liu D., Wu W., Jiang H. (2016). Transition metal free intermolecular direct oxidative C–N bond formation to polysubstituted pyrimidines using molecular oxygen as the sole oxidant. J. Org. Chem..

[14] Zhan J.-L., Wu M.-W., Chen F., Han B. (2016). Cu-catalyzed [3 + 3] annulation for the synthesis of pyrimidines via β-C(sp3)–H functionalization of saturated ketones. J. Org. Chem..

[15] Chu X.-Q., Cao W.-B., Xu X.-P., Ji S.-J. (2017). Iron catalysis for modular pyrimidine synthesis through β-ammoniation/cyclization of saturated carbonyl compounds with amidines. J. Org. Chem..

[16] Kolomoitsev O.O., Gladkov E.S., Kotlyar V.M., Pedan P.I., Onipko O.V., Buravov O.V., Chebanov V.A. (2020). Efficient synthesis of imidazole and pyrimidine derivatives. Chem. Heterocycl. Compd..

[17] Lou Z., Zhang X. (2010). Protein targets for structure-based anti-mycobacterium tuberculosis drug discovery. Protein Cell.

[18] Jackson M., McNeil M.R., Brennan P.J. (2013). Progress in targeting cell envelope biogenesis in *Mycobacterium Tuberculosis*. Future Microbiol..

[19] Mdluli K., Kaneko T., Upton A. (2014). Tuberculosis drug discovery and emerging targets. Ann. N. Y. Acad. Sci..

[20] Batt S.M., Jabeen T., Bhowruth V., Quill L., Lund P.A., Eggeling L., Alderwick L.J., Fütterer K., Besra G.S. (2012). Structural basis of inhibition of *Mycobacterium Tuberculosis* dpre1 by benzothiazinone inhibitors. Proc. Natl. Acad. Sci. USA.

[21] Goodsell D.S., Morris G.M., Olson A.J. (1996). Automated docking of flexible ligands: Applications of autodock. J. Mol. Recognit..

[22] Trott O., Olson A.J. (2010). Autodock vina: Improving the speed and accuracy of docking with a new scoring function, efficient optimization, and multithreading. J. Comput. Chem..

[23] Ali M.T., Blicharska N., Shilpi J.A., Seidel V. (2018). Investigation of the anti-TB potential of selected propolis constituents using a molecular docking approach. Sci. Rep..

[24] Li P., Wang B., Fu L., Guo K., Ma C., Wang B., Lin Z., Li G., Huang H., Lu Y. (2021). Identification of novel benzothiopyranones with ester and amide motifs derived from active metabolite as promising leads against *Mycobacterium Tuberculosis*. Eur. J. Med. Chem..

[25] Hong W., Wang Y., Chang Z., Yang Y., Pu J., Sun T., Kaur S., Sacchettini J.C., Jung H., Lin Wong W. (2015). The identification of novel *Mycobacterium Tuberculosis* DHFR inhibitors and the investigation of their binding preferences by using molecular modelling. Sci. Rep..

[26] Raimondi M.V., Randazzo O., La Franca M., Barone G., Vignoni E., Rossi D., Collina S. (2019). Dhfr inhibitors: Reading the past for discovering novel anticancer agents. Molecules.

[27] Sharma K., Neshat N., Sharma S., Giri N., Srivastava A., Almalki F., Saifullah K., Alam M.M., Shaquiquzzaman M., Akhter M. (2020). Identification of novel selective *Mtb*-DHFR inhibitors as antitubercular agents through structure-based computational techniques. Arch. Pharm..

[28] Li R., Sirawaraporn R., Chitnumsub P., Sirawaraporn W., Wooden J., Athappilly F., Turley S., Hol W.G.J. (2000). Three-dimensional structure of *M. Tuberculosis* dihydrofolate reductase reveals opportunities for the design of novel tuberculosis drugs. J. Mol. Biol..

[29] Wróbel A., Baradyn M., Ratkiewicz A., Drozdowska D. (2021). Synthesis, biological activity, and molecular dynamics study of novel series of a trimethoprim analogs as multi-targeted compounds: Dihydrofolate reductase (DHFR) inhibitors and DNA-binding agents. Int. J. Mol. Sci..

[30] Bhutani I., Loharch S., Gupta P., Madathil R., Parkesh R. (2015). Structure, dynamics, and interaction of *Mycobacterium Tuberculosis* (*Mtb*) DprE1 and DprE2 examined by molecular modeling, simulation, and electrostatic studies. PLoS ONE.

[31] Makarov V., Lechartier B., Zhang M., Neres J., van der Sar A.M., Raadsen S.A., Hartkoorn R.C., Ryabova O.B., Vocat A., Decosterd L.A. (2014). Towards a new combination therapy for tuberculosis with next generation benzothiazinones. EMBO Mol. Med..

[32] Borges de Melo E., da Silveira Gomes A., Carvalho I. (2006). A- and β-glucosidase inhibitors: Chemical structure and biological activity. Tetrahedron.

[33] Li T., Guo L., Zhang Y., Wang J., Zhang Z., Li J., Zhang W., Lin J., Zhao W., Wang P.G. (2011). Structure–activity relationships in a series of C2-substituted gluco-configured tetrahydroimidazopyridines as β-glucosidase inhibitors. Bioorg. Med. Chem..

[34] Michelin K., Wajner A., Goulart L.d.S., Fachel Â.A., Pereira M.L.S., de Mello A.S., Souza F.T.S., Pires R.F., Giugliani R., Coelho J.C. (2004). Biochemical study on β-glucosidase in individuals with gaucher’s disease and normal subjects. Clinica Chim. Acta.

[35] Chen G.-Y., Zhang H., Yang F.-Q. (2021). A simple and portable method for β-glucosidase activity assay and its inhibitor screening based on a personal glucose meter. Analyt. Chim. Acta.

[36] Kazmi M., Zaib S., Ibrar A., Amjad S.T., Shafique Z., Mehsud S., Saeed A., Iqbal J., Khan I. (2018). A new entry into the portfolio of α-glucosidase inhibitors as potent therapeutics for type 2 diabetes: Design, bioevaluation and one-pot multi-component synthesis of diamine-bridged coumarinyl oxadiazole conjugates. Bioorg. Chem..

[37] Riaz S., Khan I.U., Yar M., Ashraf M., Rehman T.U., Shaukat A., Jamal S.B., Duarte V.C.M., Alves M.J. (2014). Novel pyridine-2,4,6-tricarbohydrazide derivatives: Design, synthesis, characterization and in vitro biological evaluation as α- and β-glucosidase inhibitors. Bioorg. Chem..

[38] Parizadeh H., Garampalli R.H. (2016). Evaluation of some *lichen* extracts for β-glucosidase inhibitory as a possible source of herbal anti-diabetic drugs. Am. J. Biochem..

[39] Chu C., Deng J., Man Y., Qu Y. (2017). Green tea extracts epigallocatechin-3-gallate for different treatments. BioMed Res. Intern..

[40] Hu Z.G., Liu J., Zeng P.L., Dong Z.B. (2004). Synthesis of α, α′-bis(substituted benzylidene)ketones catalysed by a SOCl_2_/EtOH reagent. J. Chem. Res..

[41] Lüth A., Löwe W. (2008). Syntheses of 4-(indole-3-yl)quinazolines—A new class of epidermal growth factor receptor tyrosine kinase inhibitors. Eur. J. Med. Chem..

[42] Gundla R., Kazemi R., Sanam R., Muttineni R., Sarma J.A.R.P., Dayam R., Neamati N. (2008). Discovery of novel small-molecule inhibitors of human epidermal growth factor receptor-2: Combined ligand and target-based approach. J. Med. Chem..

