# Supplementary material for: Synthesis of Novel Derivatives of 5,6,7,8-Tetrahydroquinazolines Using α-Aminoamidines and In Silico Screening of Their Biological Activity
Source: Int J Mol Sci. 2022 Mar 29;23(7):3781. doi: 10.3390/ijms23073781 (PMC8999073; doi:10.3390/ijms23073781)
Supplement: Supplementary file 1 [file ijms-23-03781-s001.zip › ijms-1504750-supplementary.pdf]

# Supplementary Information

for

# Synthesis of Novel Derivatives of 5,6,7,8-Tetrahydroquinazolines using of α-Aminoamidines and *In Silico* Screening of Their Biological Activity

Arsenii D. Snizhko[1], Alexander V. Kyrychenko[1], Eugene S. Gladkov[1,2*]

[1] *V. N. Karazin Kharkiv National University, Institute of Chemistry and School of Chemistry, 4 Svobody Sq., Kharkiv 61022, Ukraine*
*e-mail: eugenegladkov@gmail.com*
[2] *State Scientific Institution "Institute for Single Crystals", National Academy of Sciences of Ukraine, 60 Nauky Ave., Kharkiv 61072, Ukraine*

## Mass-Spectra, 1H and 13C NMR spectra for compounds 3a-g and 4e-g

**Table of contents**

1. $^{1}$H and $^{13}$C NMR spectra…………………….............................................. S2
2. Mass Spectra ……………….................................................................... S12

## 1. $^{1}H$ and $^{13}C$ NMR spectra

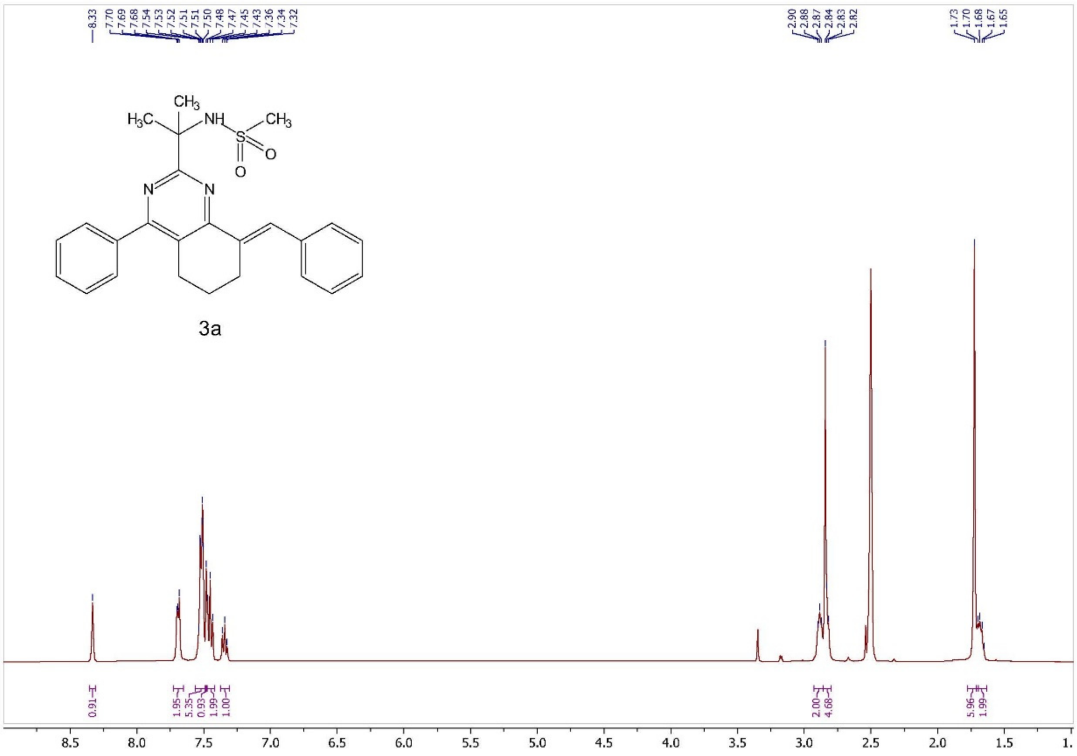

**Figure S1.** $^{1}$H NMR of compound 3a

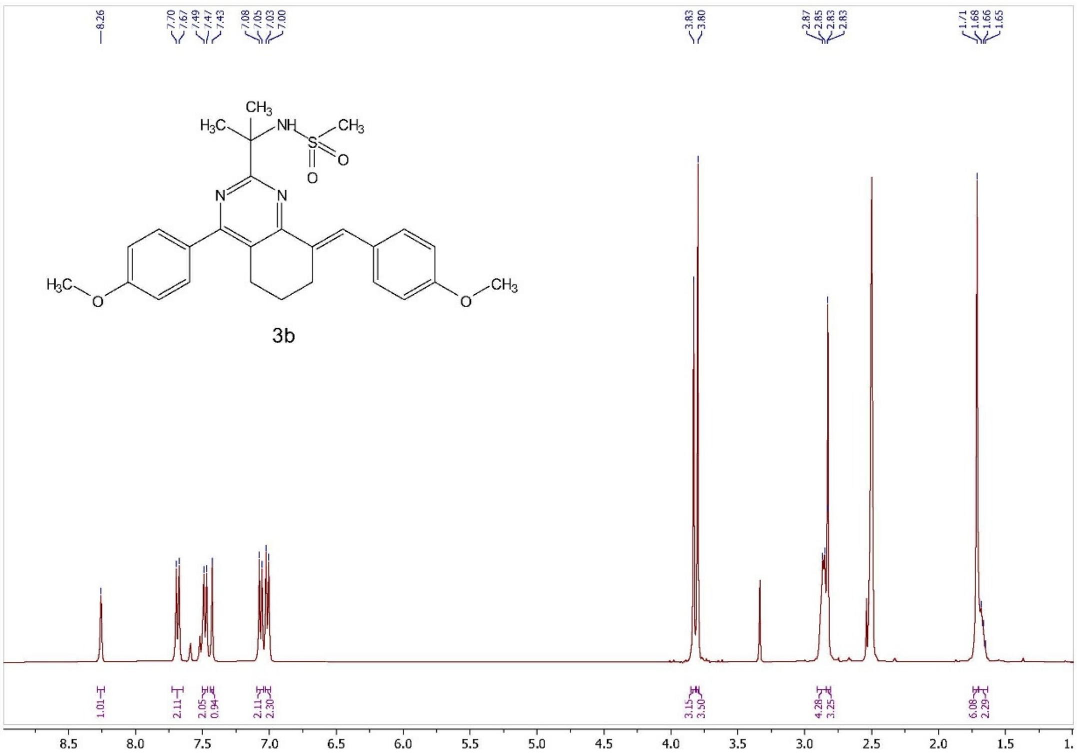

**Figure S2.** $^{1}$H NMR of compound 3b

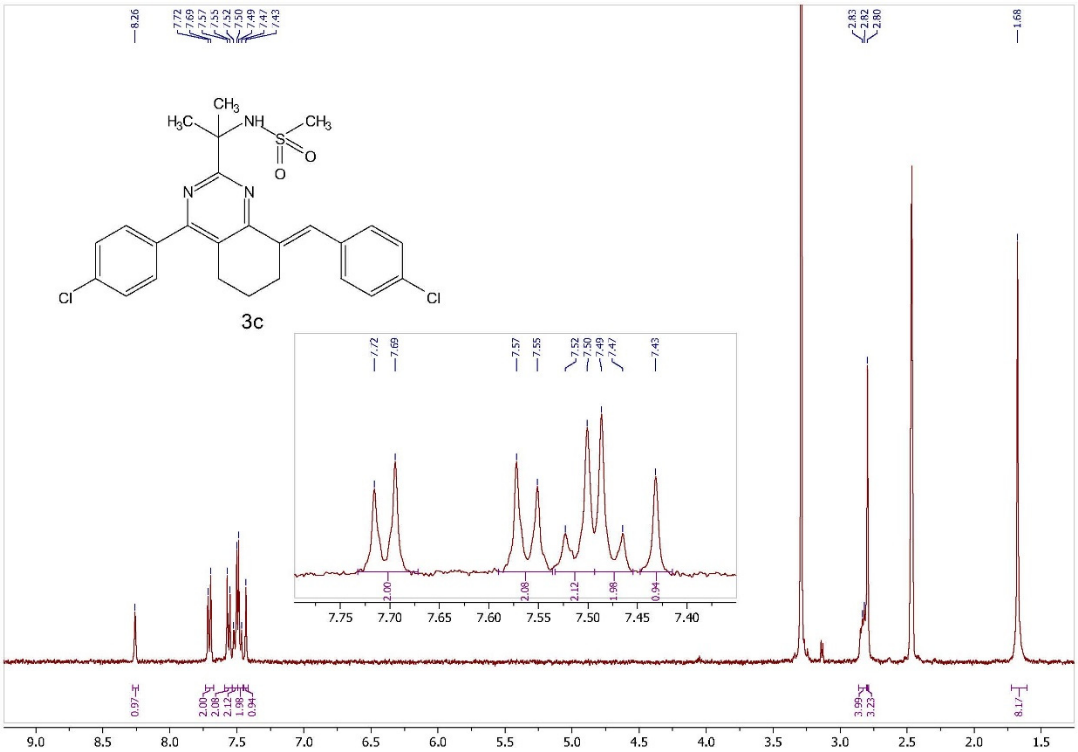


**Figure S3.** $^1$H NMR of compound 3c

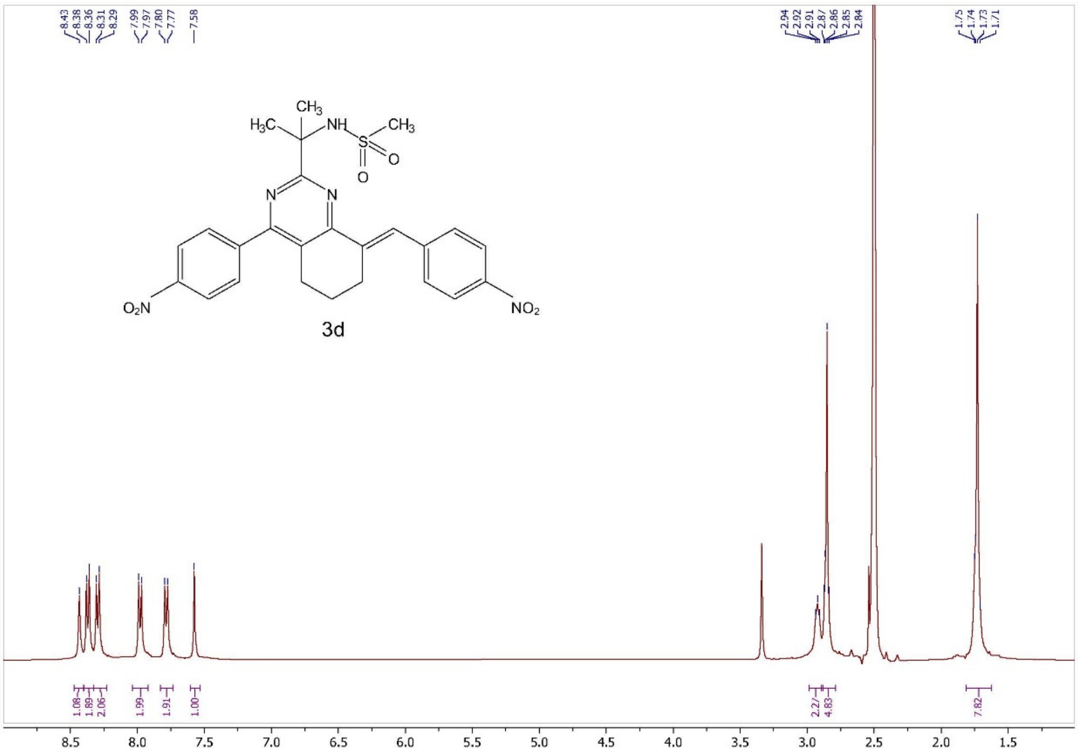


**Figure S4.** $^1$H NMR of compound 3d

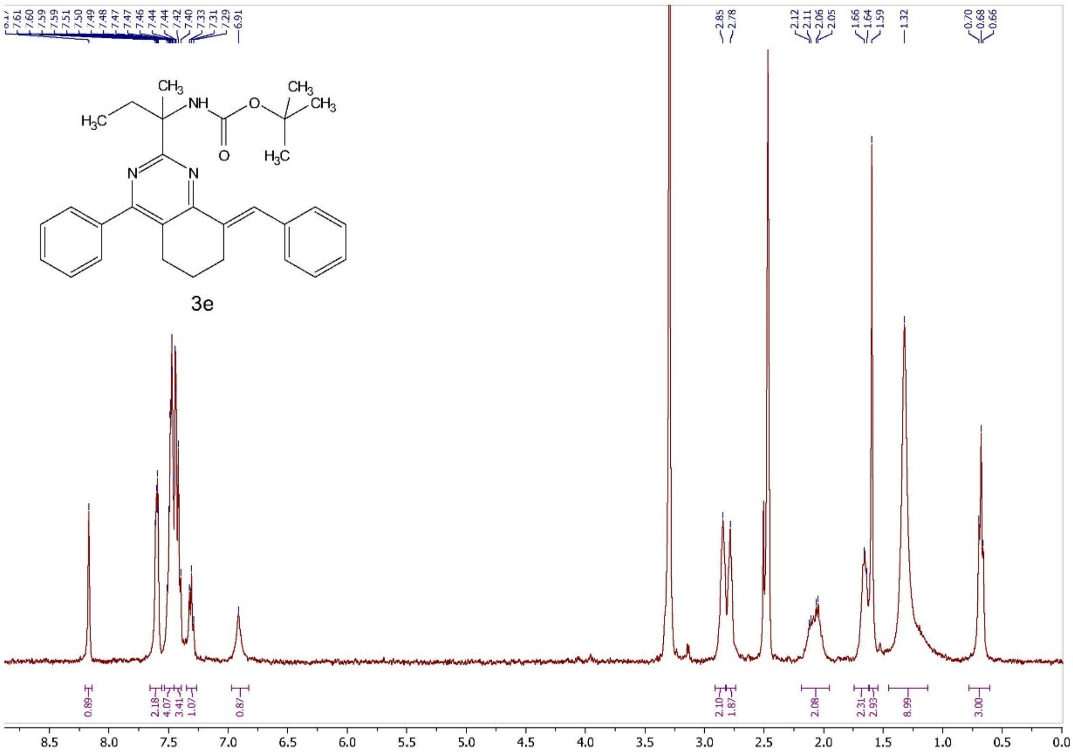

**Figure S5.** $^1$H NMR of compound 3e

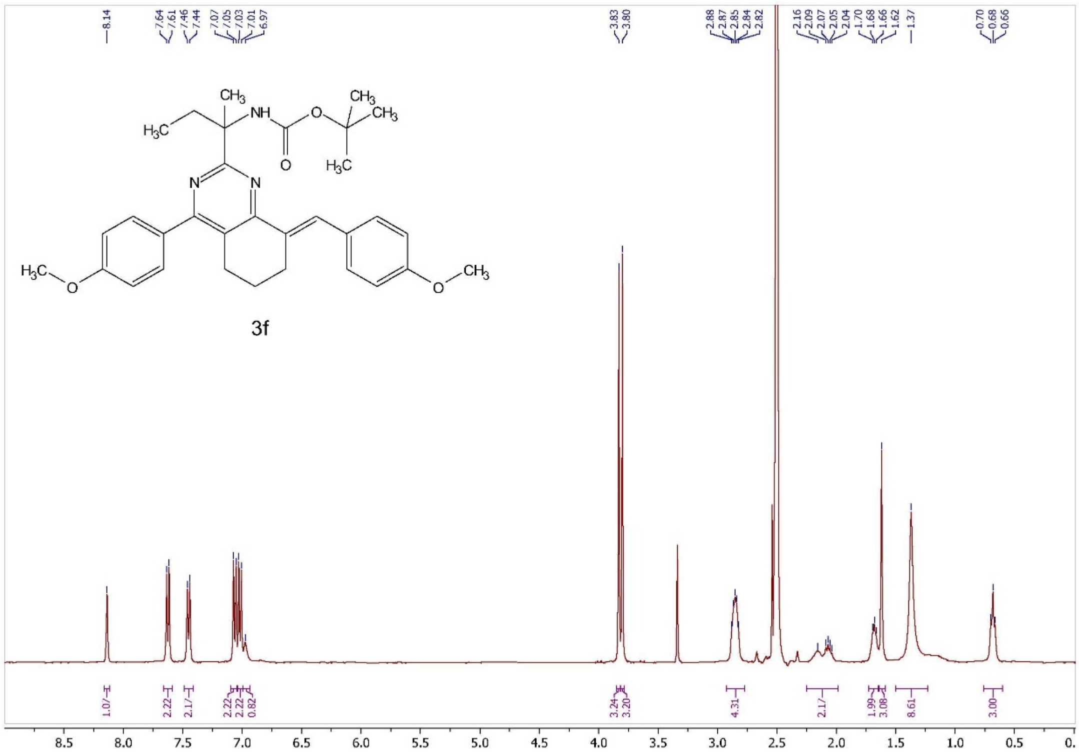

**Figure S6.** $^1$H NMR of compound 3f

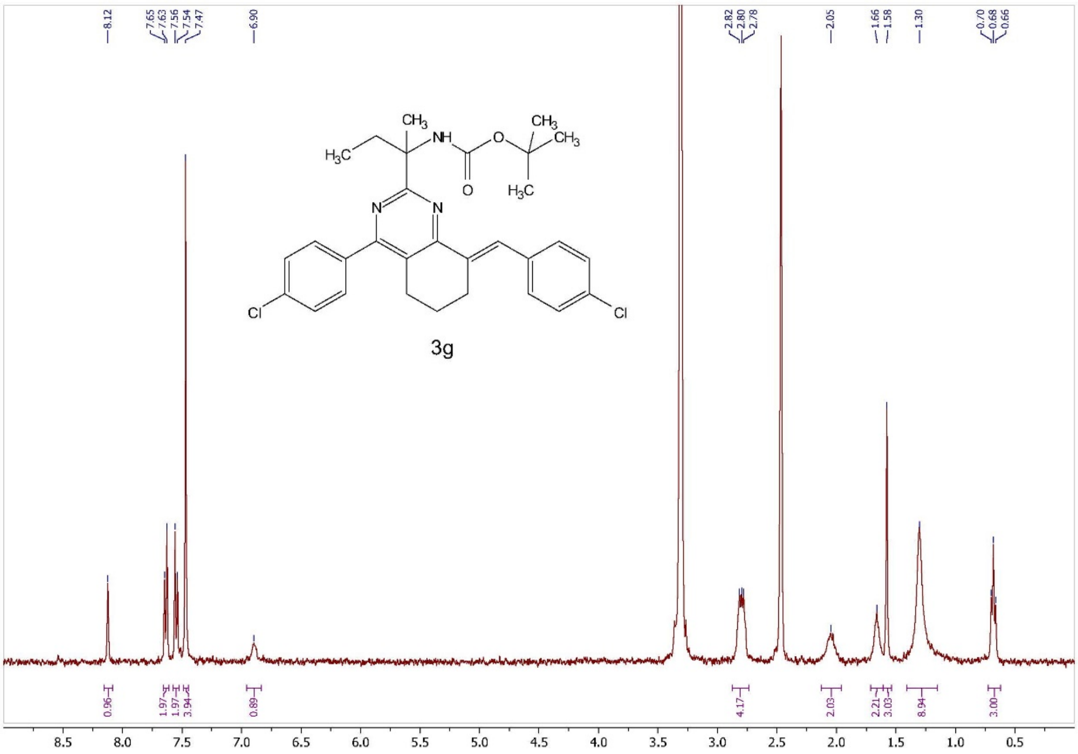

**Figure S7.** $^{1}$H NMR of compound 3g

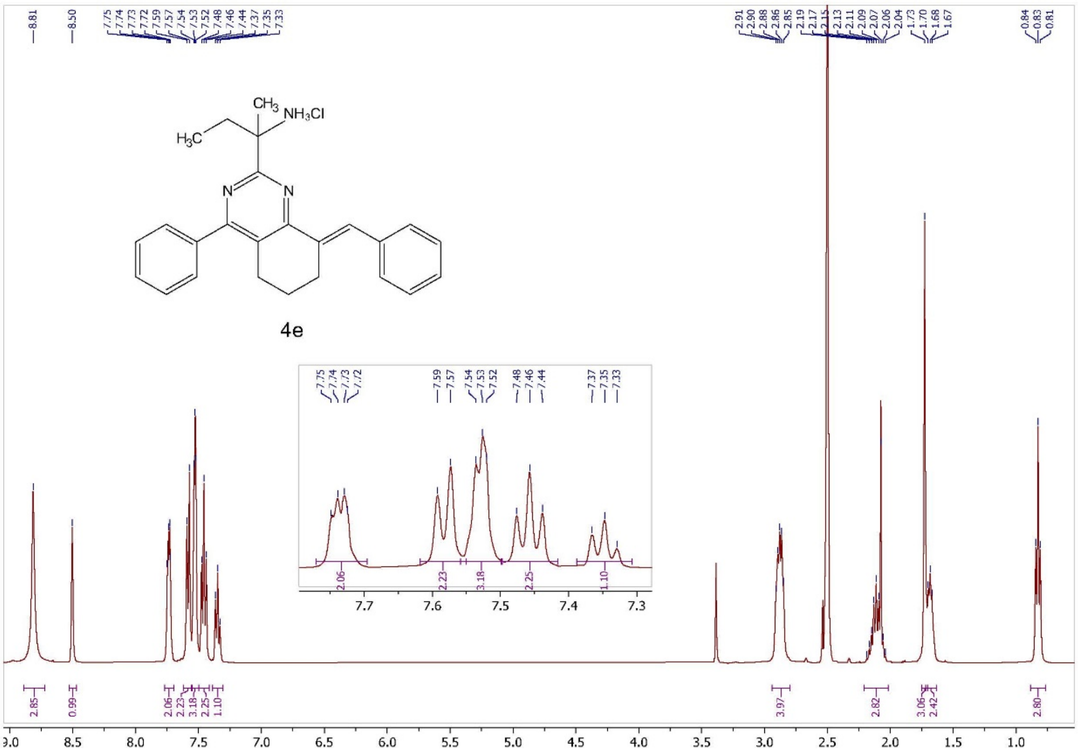

**Figure S8.** $^{1}$H NMR of compound 4e

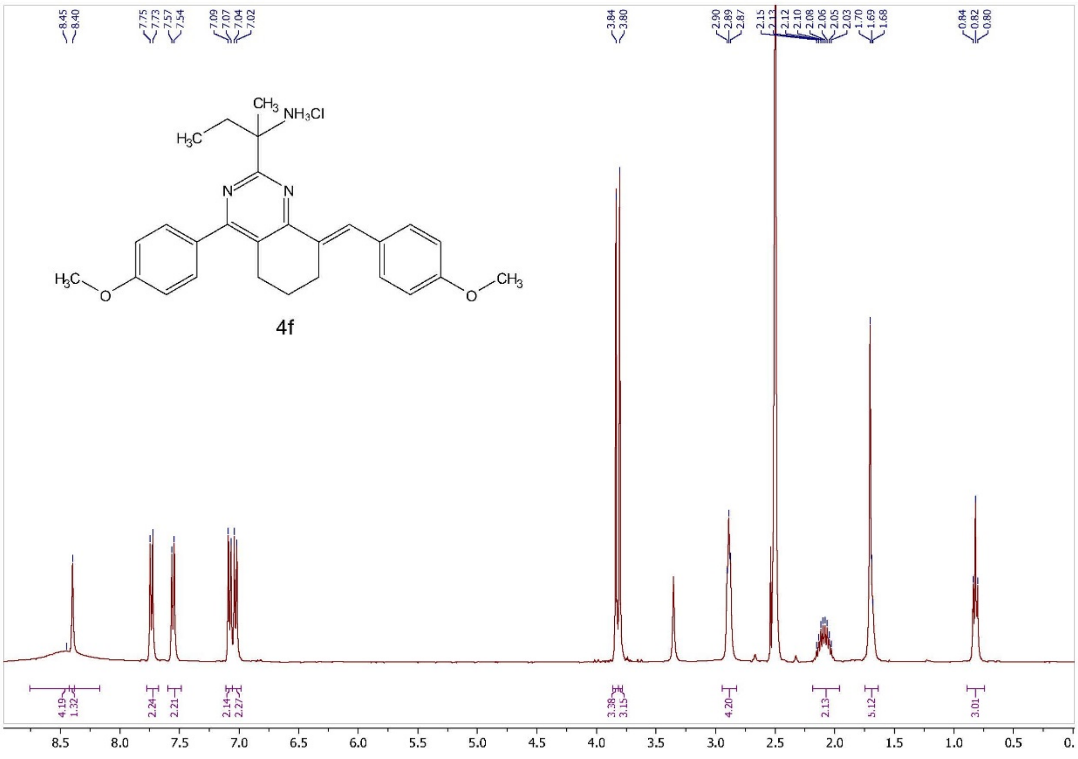

**Figure S9.** $^{1}$H NMR of compound 4f

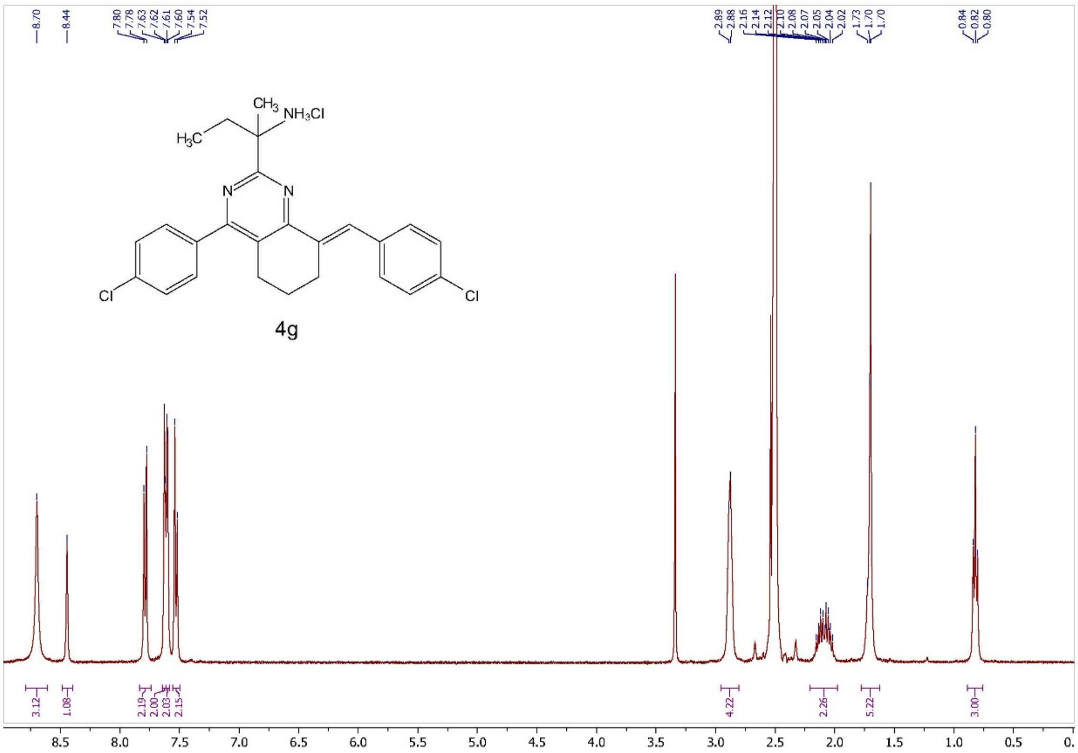

**Figure S10.** $^{1}$H NMR of compound 4g

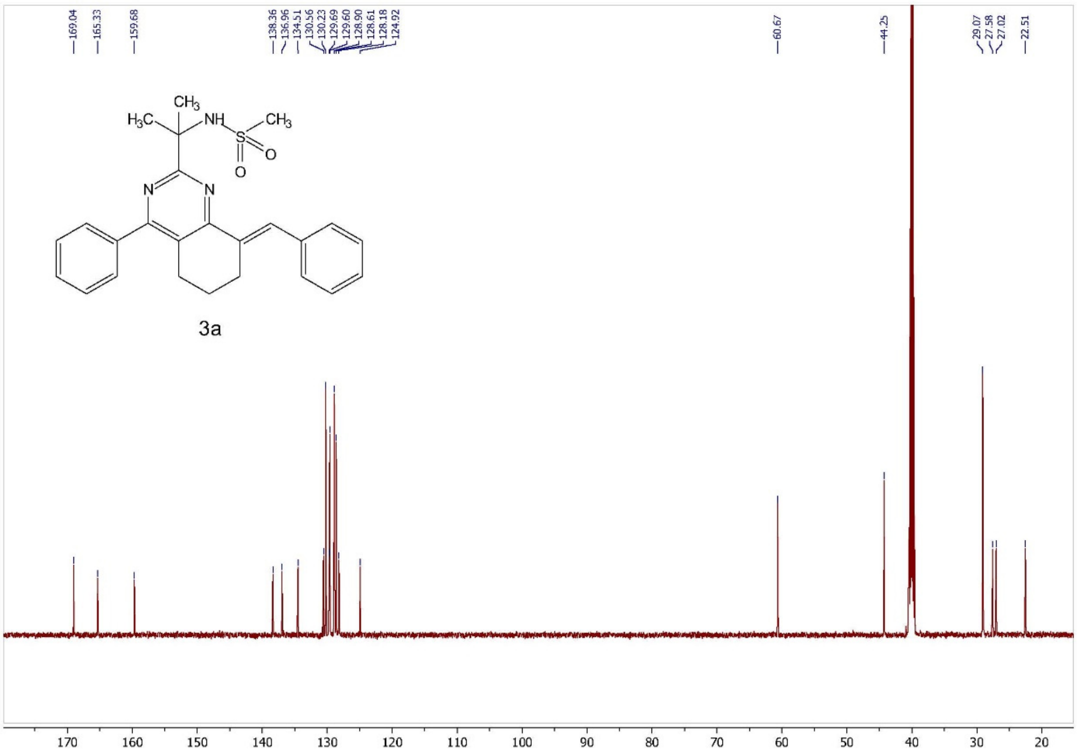


**Figure S11.** $^{13}$C NMR of compound 3a

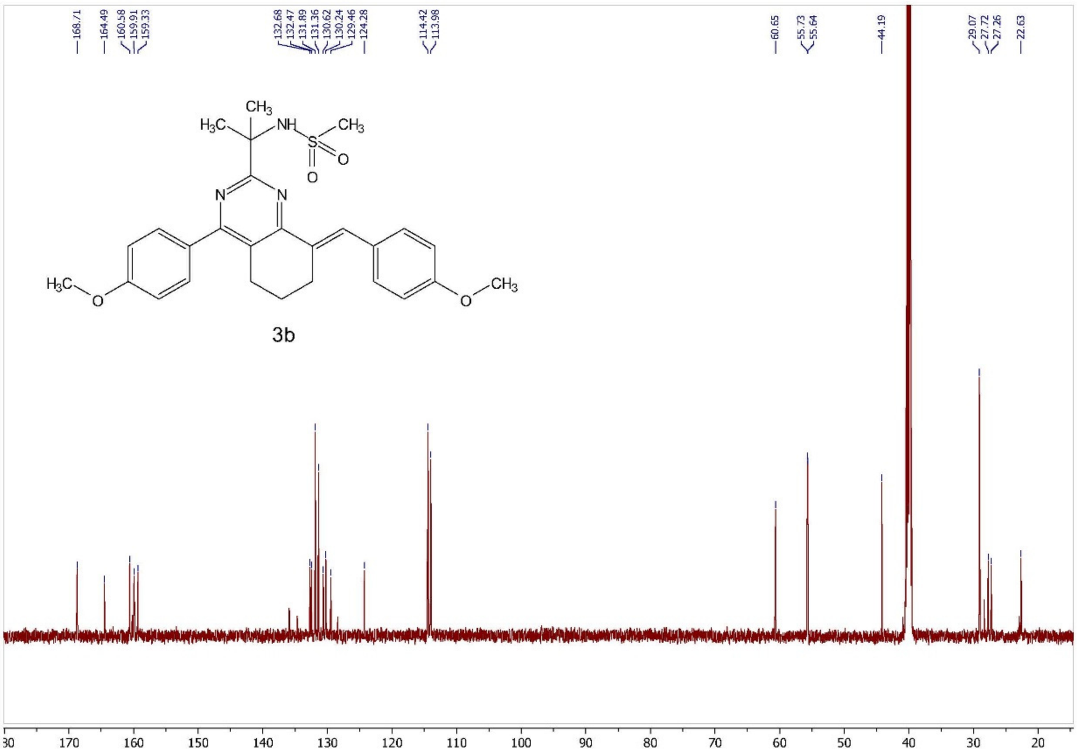


**Figure S12.** $^{13}$C NMR of compound 3b

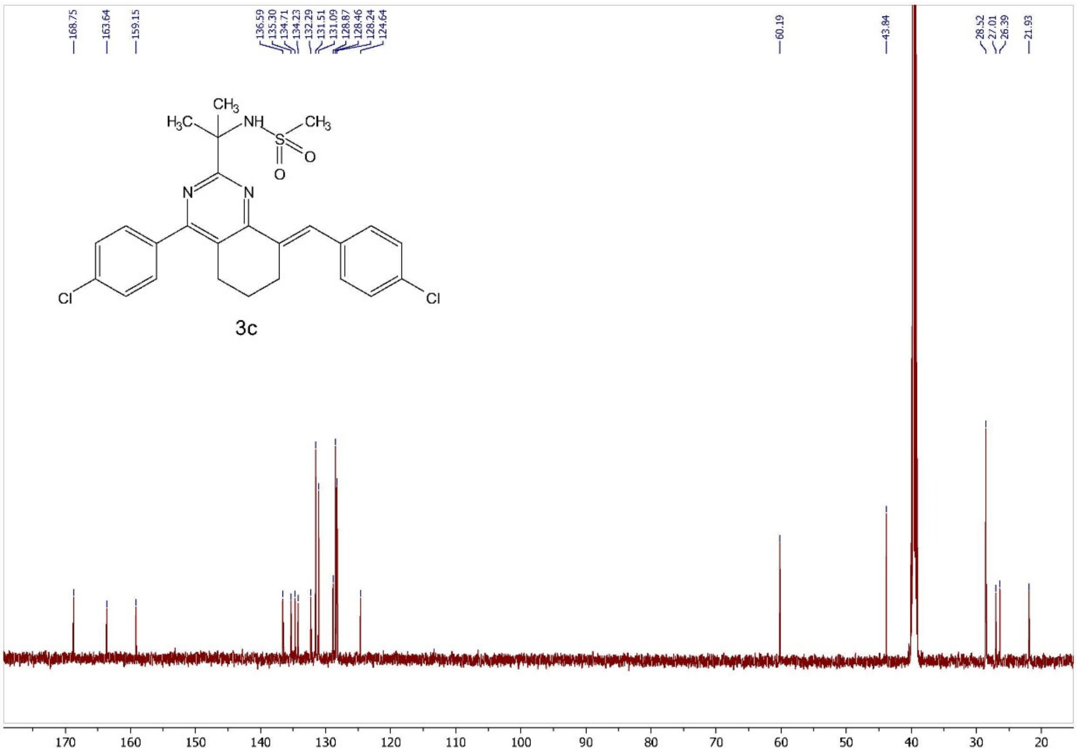


**Figure S13.** $^{13}$C NMR of compound 3c

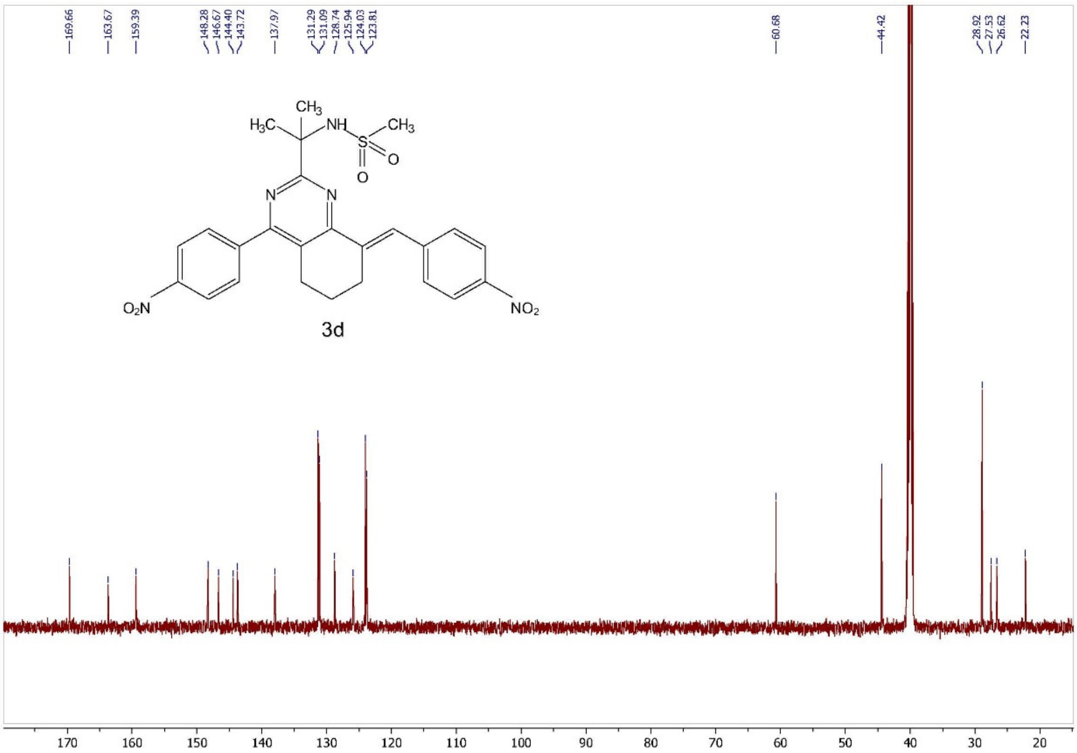


**Figure S14.** $^{13}$C NMR of compound 3d

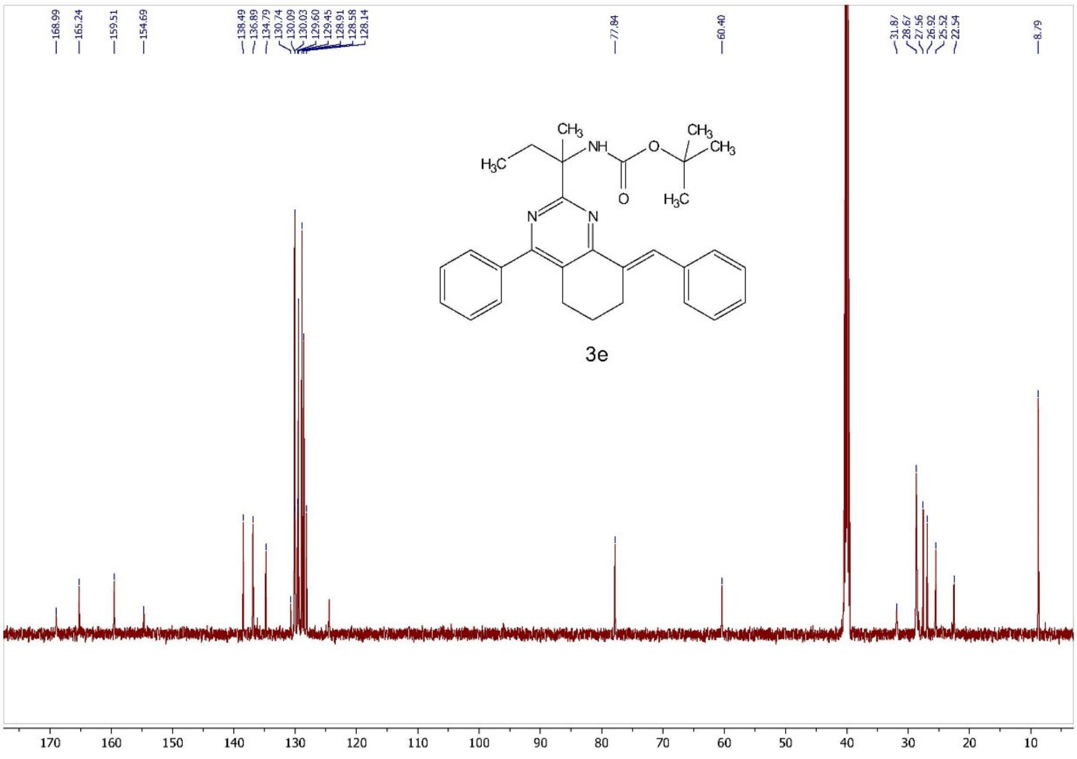

**Figure S15.** $^{13}$C NMR of compound 3e

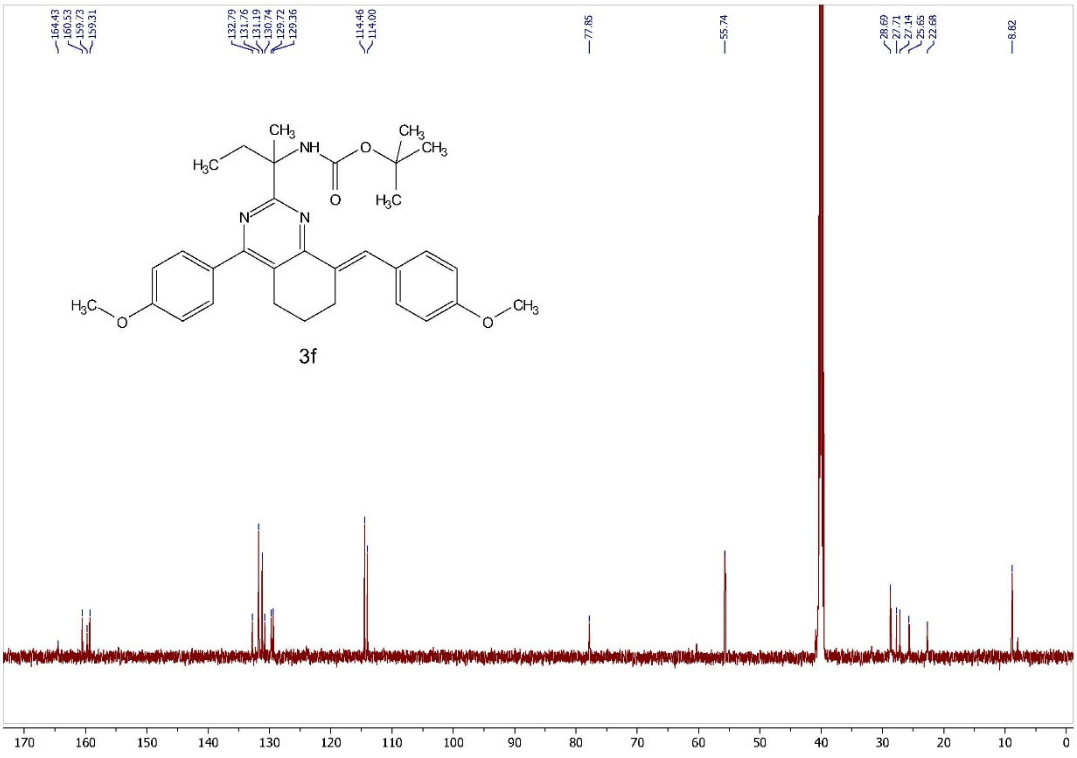

**Figure S16.** $^{13}$C NMR of compound 3f

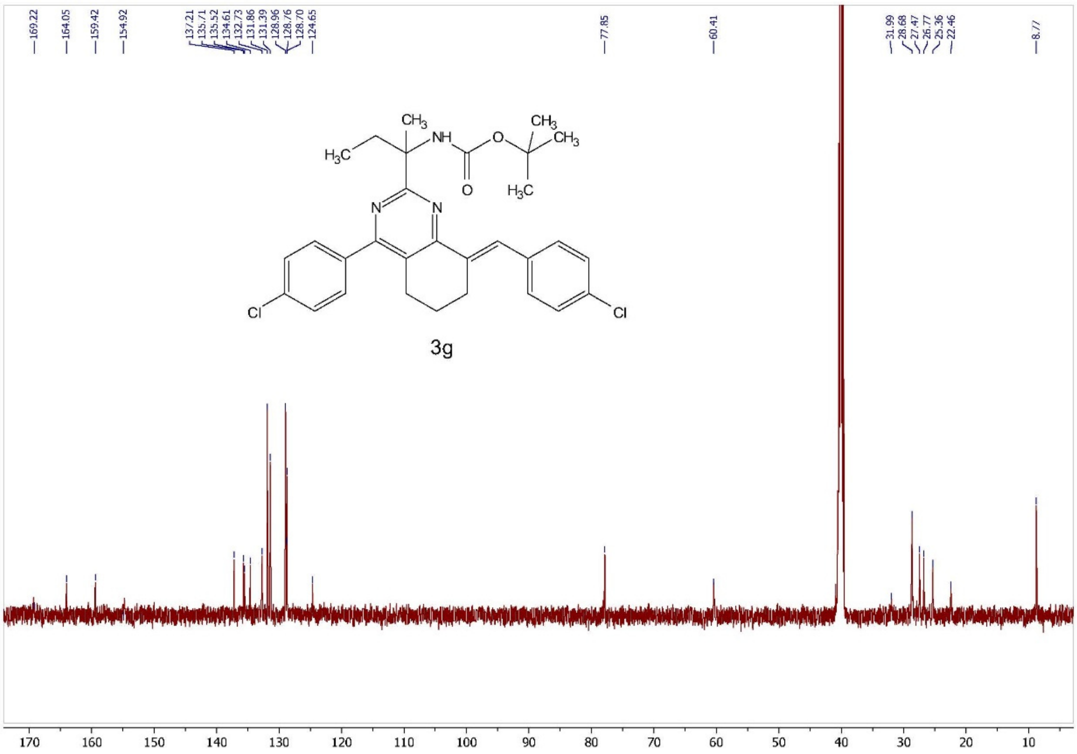

**Figure S17.** $^{13}$C NMR of compound 3g

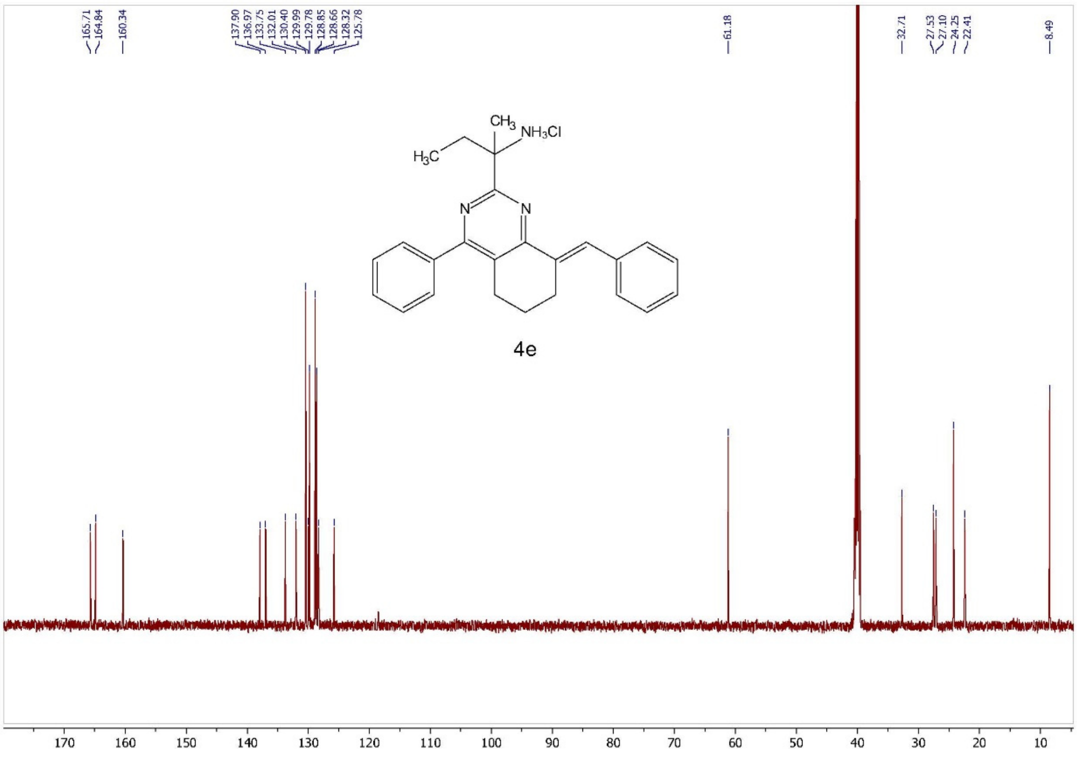

**Figure S18.** $^{13}$C NMR of compound 4e

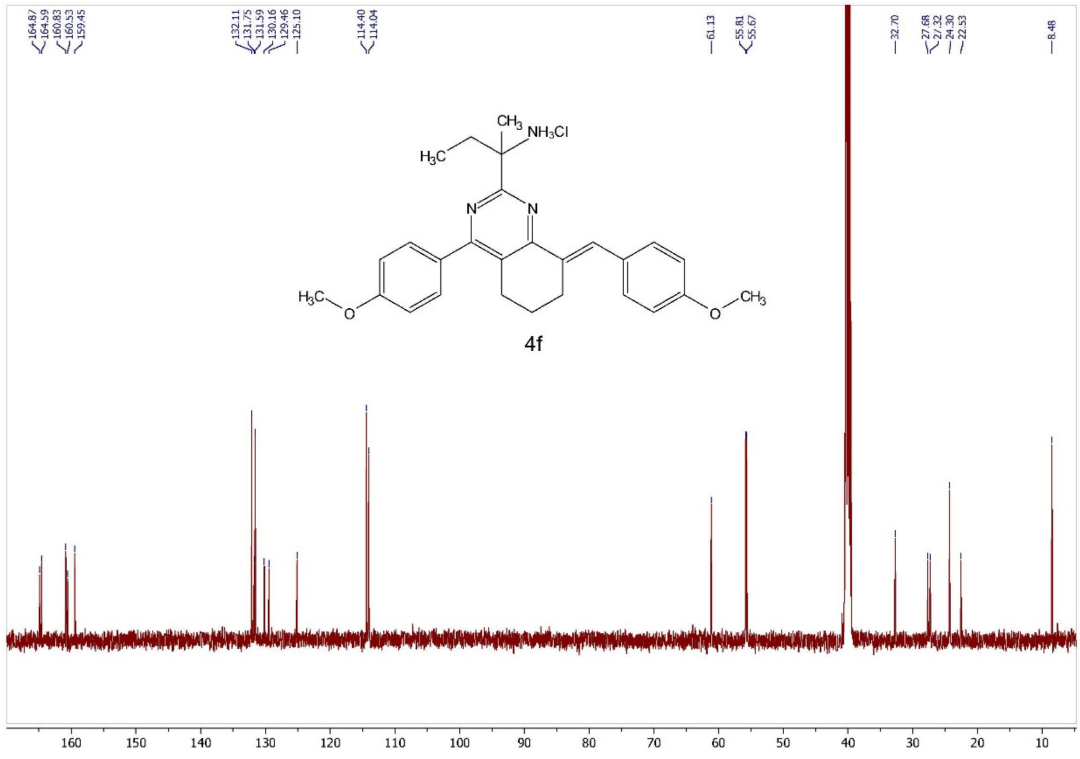

**Figure S19.** $^{13}$C NMR of compound 4f

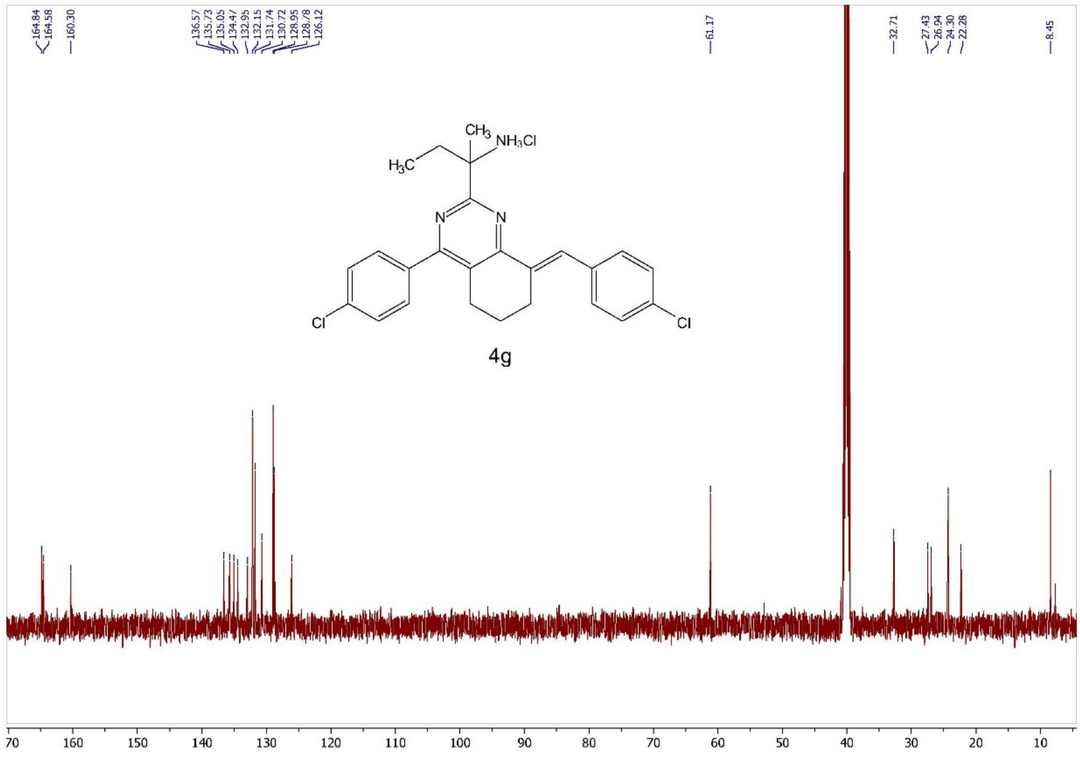

**Figure S20.** $^{13}$C NMR of compound 4g

## 2. Mass Spectra (MS)

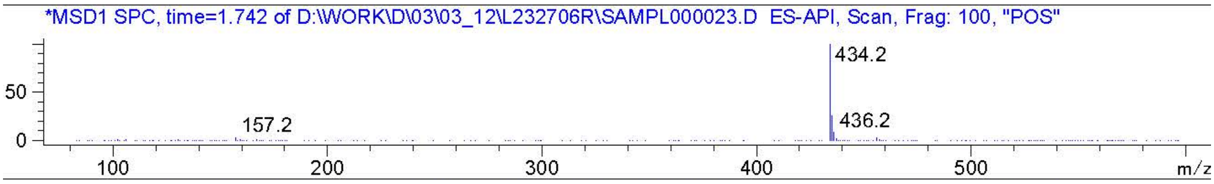


**Figure S21.** MS of compound 3a

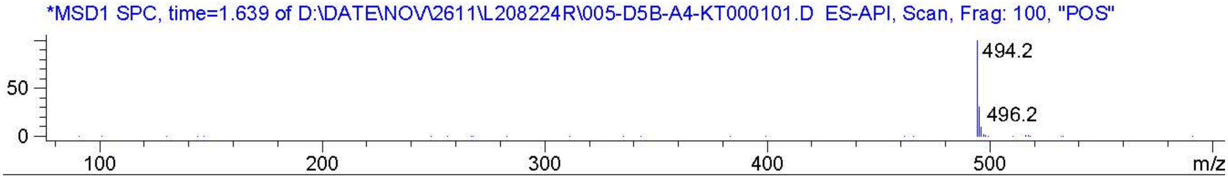


**Figure S22.** MS of compound 3b

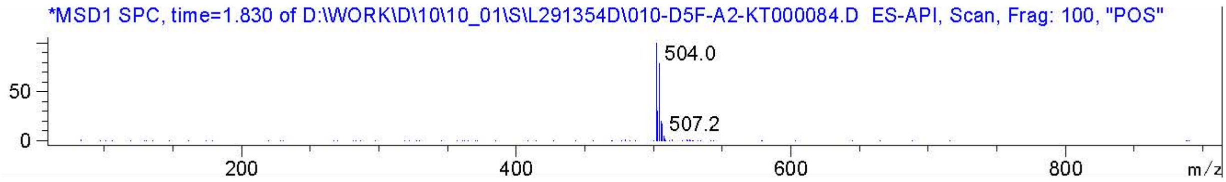


**Figure S23.** MS of compound 3c

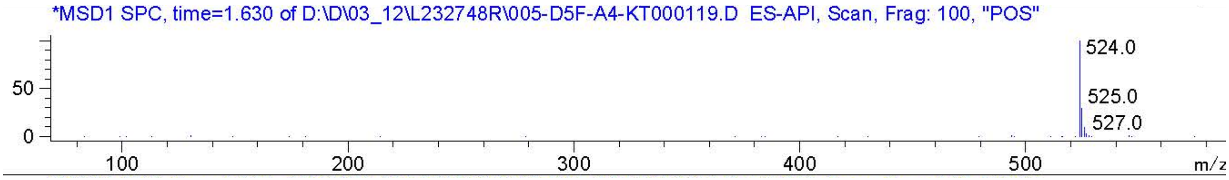


**Figure S24.** MS of compound 3d

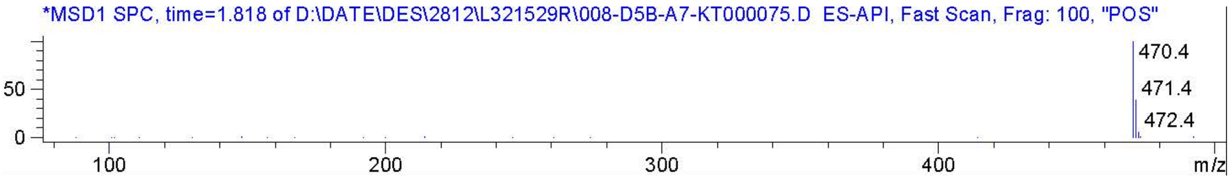


**Figure S25.** MS of compound 3e

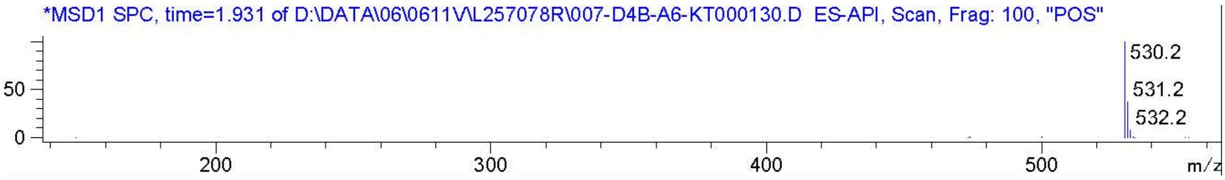


**Figure S26.** MS of compound 3f

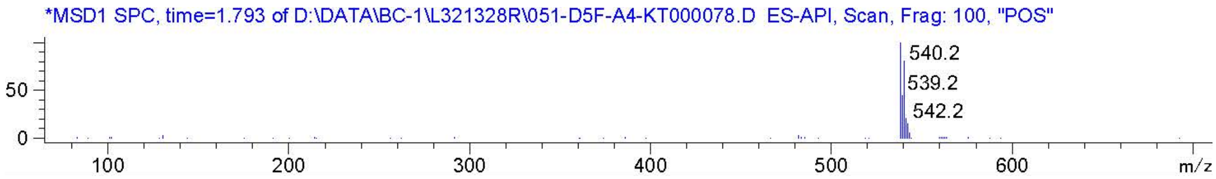


**Figure S27.** MS of compound 3g

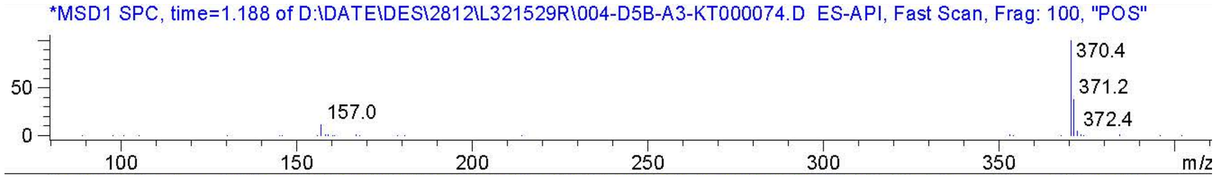


**Figure S28.** MS of compound 4e

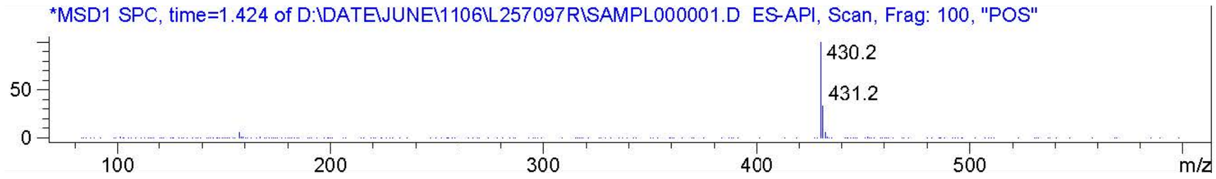


**Figure S29.** MS of compound 4f

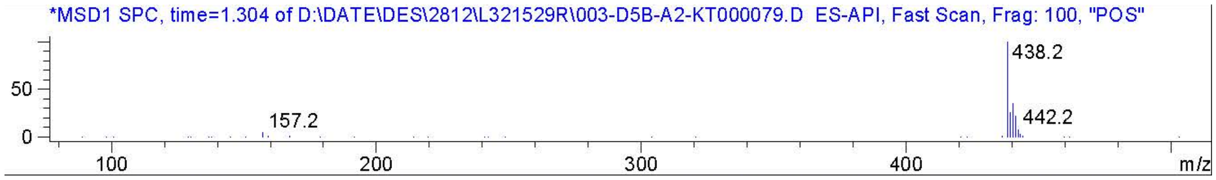


**Figure S30.** MS of compound 4g
